# Supplementary material for: The nematode Caenorhabditis elegans has a cationic amphiphilic drug (CAD) defense system
Source: PLoS Biol. 2026 Sep 21;24(9):e3004006. doi: 10.1371/journal.pbio.3004006 (PMC13619054; doi:10.1371/journal.pbio.3004006)
Supplement: Supplementary file 1 [file pbio.3004006.s014.pdf]

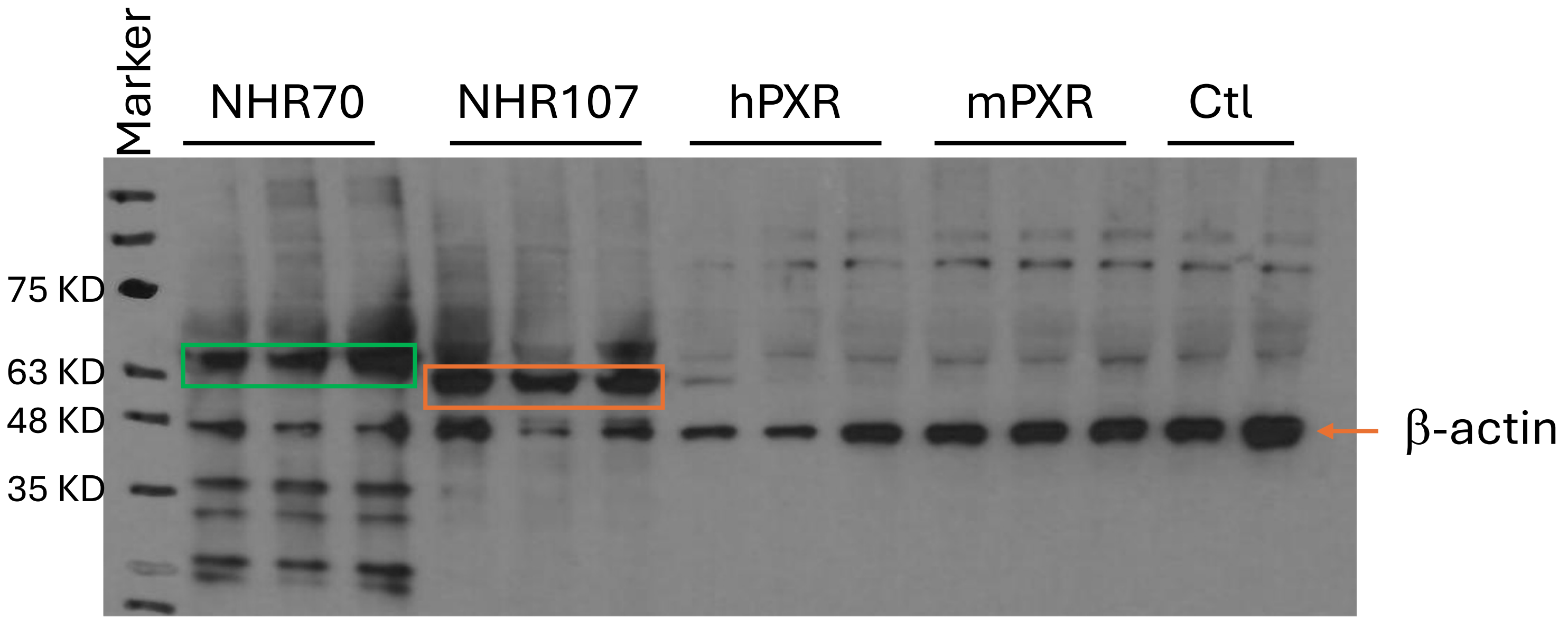

Marker
NHR70
NHR107
hPXR
mPXR
Ctl
75 KD
63 KD
48 KD
35 KD
β-actin
