# Supplementary material for: The nematode Caenorhabditis elegans has a cationic amphiphilic drug (CAD) defense system
Source: PLoS Biol. 2026 Sep 21;24(9):e3004006. doi: 10.1371/journal.pbio.3004006 (PMC13619054; doi:10.1371/journal.pbio.3004006)
Supplement: Supplementary file 2 [file pbio.3004006.s017.pdf]

CAD Manuscript Reviews from PLoS Biology

Editorial Comments

Thank you for your patience while your manuscript entitled "A Cationic Amphiphilic Drug (CAD) Defense System in the Nematode Caenorhabditis elegans" was peer-reviewed at PLOS Biology. Please also accept my apologies for the delay in sending you our decision. The manuscript has now been evaluated by the PLOS Biology editors, an Academic Editor with relevant expertise, and by three independent reviewers.

The reviews are attached below. As you will see, the reviewers find the conclusions novel and interesting, but they also raise several issues that will need to be addressed before we can consider the manuscript for publication. Reviewer 1 thinks that **a ligand binding essay should be performed to confirm CAD binding to NHR-70 and NHR-107 to strengthen the study**. This reviewer also requests **an additional assay to confirm the experiments with lysosomal acidity using cDCFDA,** among other issues.

Reviewer 2 is mostly satisfied and only raises minor points, whereas Reviewer 3 notes that parts of the study are a bit hard to follow, thus the paper would benefit from some rewriting to make it more accessible for a wide audience. In addition, the reviewer suggests that a cartoon could be used to explain the model better.

In light of the reviews, which you will find at the end of this email, we would like to invite you to revise the work to thoroughly address the reviewers' reports. Given the extent of revision needed, we cannot make a decision about publication until we have seen the revised manuscript and your response to the reviewers' comments. Your revised manuscript is likely to be sent for further evaluation by all or a subset of the reviewers.

Authors' Opening Remarks: We thank the editors and the reviewers for considering our manuscript, which is the product of the works of both an entire MSc and and entire PhD degree, along with the generous efforts of our collaborators. Below, we answer each of the queries in detail.

Reviewers' comments

Reviewer #1-Opening Remarks: This study identifies a defence system in Caenorhabditis elegans that protects against cationic amphiphilic drugs (CADs), which can cause lysosomal dysfunction. Using a combination of genetics, transcriptomics, metabolomics, chemoinformatics, and structural modeling, the authors provide compelling evidence that nuclear receptors NHR 70 and NHR 107 regulate CYP 35B enzymes and PGP 13/PGP 14 transporters to detoxify CADs. The authors provide evidence for CYP 35Bs metabolizing CADs and PGP 13/14 exporting metabolites, both critical for reducing drug-induced toxicity. A pgp 13 fluorescent reporter enabled them to screen a compound library, identifying CAD-like compounds consistent with computational predictions. They show that the defence system is also activated by certain Streptomyces strains, hinting at its evolutionary purpose. While some mechanisms remain unclear, in general, the study is well designed and executed, have several elements of novelty and will be of broad interest to the scientific community.
Author's Response: We thank the reviewer for their detailed assessment and kind words.

Reviewer #1-Comment #1: One of the main drawbacks of the study is the lack of direct evidence of CAD binding to NHR-70 and NHR-107. Direct evidence in form of a ligand binding assay would strengthen their study.
Authors' Response: The issue of whether the CADs are ligands for NHR-70 and NHR-107 is an issue that we studied earlier in the project and which yielded ambiguous results. We returned to these types of experiments again and tried different approaches and still came up with ambiguous answers. We'll explain two of these approaches here.

First, we returned to cell-based experiments where we heterologously express nuclear receptor (NR) ligand-binding domain plus linker fusions to GAL4 DNA-binding domain (NR LBD::GAL4DBD) in the background of a luciferase reporter with GAL binding sites, which has been employed routinely for mammalian NR-ligand tests (Liu *et al.* 2024). We previously used HEK293 cells, but have since learned that HepG2s provide larger signal-to-noise ratios, so we repeated those experiments with HepG2s. Our positive control was human PXR LBD::GAL4DBD with its well characterized ligand SR12813 (Motta *et al.* 2018). **Appendix A** at the end of this document describes these experiments in detail.

In summary, the positive control worked well, yielding 50-100 fold induction of reporter expression, while the experimentals yielded no obvious signal, despite good expression of the fusion proteins (**Appendix A**). It is important to note that of the 284 NRs in *C. elegans*, only one has been shown to directly interact with its ligand using this traditional heterologous assay (Arda *et al.* 2010), and a co-author from that work (Cummins) is also a co-author on this paper and has been involved in these experiments.

In addition to the possibility that the CADs do not interact directly with the NRs, there may be many reasons why these experiments failed to demonstrate an interaction between the CADs and the NRs, including the possibility that necessary *C. elegans*-specific co-factors that mediate the interaction between ligand-bound NRs and RNA polymerase II are absent from the human-derived cell lines.

The second approach that we took to investigate whether the CADs are likely direct interactors with NHR-70 and NHR-107 was to use computational docking, which was led by our

computational specialist collaborator David Koes. We created poses with AlphaFold 3 and separately with Boltz-2 based on crystal structures where available (for PXR and VDR controls), and used GNINA for docking.

We established three benchmarks to determine whether the docking was yielding useful information, and if so, whether the interactions are as predicted. In summary, the positive (PXR and known ligands SR12813 and hyperforin) and negative (VDR and ligands SR12813 and hyperforin) controls behaved somewhat as expected, but the magnitude of the differences were expected to be greater (**Appendix B**). Despite this, there was no evidence that supported the claim that the CADs directly interact with the NHR-70 and NHR-107 ligand binding domains (**Appendix B**).

Finally, we conducted pilot experiments for direct interactions using bacterially-expressed tagged NHR-70 and NHR-107 ligand binding domains co-incubated with the CADs, followed by immunoprecipitation and ligand detection via LC-MS, which yielded unimpressive results. Given the two sets of experiments above, we decided not to pursue these experiments further.

The experiments described above fail to provide evidence for a direct interaction, but do not prove that a direct interaction between CADs and the NRs doesn't exist. We therefore continue to frame the issue as an open question.

We note that in the original (and revised) manuscript that we explicitly state that it remains an open question as to whether the CADs directly interact with the nuclear receptors and that the information presented above changes none of our claims. In the results section where we first describe the results of the genetic screen, we write, 'The screen results suggested that CADs directly or indirectly activate the NHR-70 and NHR-107 nuclear receptors to transcriptionally up-regulate PGP-13…'. In the discussion section, the relevant paragraph begins with, 'It remains an open question as to whether CADs interact directly with the NHR-70 and NHR-107 nuclear receptors to induce a transcriptional response.'. To further reinforce this point, we added a similar sentence to the introduction, writing, 'Whether the CADs act as ligands for these nuclear receptors is an open question.'

Reviewer #1-Comment #2: Does NHR-70 and NHR-107 directly regulate the expression of the pgp genes?

Authors' Response: We emphasize that none of the claims that we make in the original or revised version is dependent on knowing whether the relevant NRs directly regulate the expression of *pgp-13* or the relevant P450s. Nonetheless, we acknowledge that this is an obvious question, and one that we have considered previously. The limited information and materials available have not led to any conclusions, however, and are thus not mentioned in the manuscript.

The most systematic approach would be to perform NHR-70 CHIPseq on animals +/- CADs. To do this, we would need either an NHR-70 antibody (which doesn't exist) or a functionally-tagged NHR-70 CRISPR line (which also doesn't exist). Once binding sites were found, we would want to test whether they are necessary to drive CAD-dependent transcription by mutating them. We simply don't have these reagents.

An alternative approach would be to use computational methods to ask whether there are TF motif matches in the corresponding promoter sequences. We consulted with our colleague and academic neighbor Tim Hughes, who is a world expert in transcription factor interactions with their DNA binding motifs (he has 65K+ citations, much of which cite his work on TF-DNA interactions). Tim's group published a candidate DNA-binding motif for NHR-70 in their Narasimhan *et al*., 2015 paper (PMID 25905672).

Tim points out that the motif is consistent with monomeric NHR binding (it was obtained using an older technology, Protein Binding Microarrays) and that there has been no subsequent validation of this motif. Moreover, nuclear receptors often work in pairs, which can alter the sequence specificity of the monomeric binding sites, and impose preferred spacing and orientation constraints. There is no motif for NHR-107.

In response to this query, Tim's group nonetheless attempted an enrichment analyses of whether the predicted NHR-70 binding motif was enriched within the promoter/enhancer regions of the CAD-responsive NHR-70-dependent genes (*pgp-13*, *cyp-35b1*, *b2*, and *b3*, and *cror-1* and *cror-2*) that are upregulated in response to 3/3 different CADs (as determined by RNA seq as discussed in the manuscript and extended by our work), as well as a larger set of genes that are up-regulated in response to 2/3 CADs, relative to a large set of negative controls.

The promoter and enhancer regions used in this attempted analysis are defined by the published work of Julie Ahringer (Janes *et al*., 2018 PMID: 30362940) based on their ATACseeq experiments, which reveal open chromatin regions that characterize active promoters (i.e. this is where the TFs would be expected to bind). We believe this is the best database of promoter sequences available. The Ahringer dataset was collected of course on non-drug treated wild type animals, and NHR-70-dependent transcription is CAD-dependent. It is therefore not surprising that 4/6 CAD/NHR-70-dependent genes are absent from the Ahringer dataset, and the two that are do not show the Narasimhan *et al* NHR-70 motif. Importantly, these two promoters may have other regions that open in the presence of CAD.

There are many other ways this analysis could be performed, but Tim suggested that ad hoc approaches (e.g. using 3 kb upstream of the ATG) would be error prone and in any case are beyond the scope of what the Hughes group could easily do at this time. He suggested running ATAC-seq on CAD-treated worms, and using more modern methods to analyze the sequence specificity of the two TFs. But again, we see this larger-scale project as beyond the scope of the current work.

Given that this exploration was inconclusive, and that it is lengthy to explain, we have elected not to include this information in the paper.

Reviewer #1-Comment #3: 76% of compounds require both NHR-70 and NHR-107 while 12% require only NHR-70. This suggests a potential combinatorial regulation. Do NHR-70 and NHR-107 form heterodimers?
Authors' Response: This would require the same set of experiments as described for comment #2 above and therefore feel that this question is beyond the scope of the manuscript. Our data <u>suggests</u> that the two NRs likely function as a heterodimer in some contexts and we cautiously

raise the possibility in the manuscript, but to investigate this *in vitro* and *in vivo* would be a large part of another graduate degree.

Reviewer #1-Comment #4: The relevance of the last section of the manuscript that deals with checking the response to Actinobacteria strains is quite vague. Out of the 65 strains, only two showed obvious increase in the pgp-13::gfp expression while all these strains are claimed to produce secondary metabolites. What are the metabolites produced by the strains 1G9 and 1E6 that induce the GFP?

Authors' Response: We regret making the definitive statement in the relevant section that, 'These strains produce diverse secondary metabolites…' and thank the reviewer for bringing that to our attention. We have modified the sentence to read, 'Many of these stains produce obvious secondary metabolites…'.

The reviewer is astute in picking up all the possible directions that our work is taking. In this case, we (Nodwell) have a student whose PhD project is exactly this. She has been working for nearly 3 years to expand our screen and identify the natural products produced by the *Actinobacteria* strains that induce expression of the CAD defense reporter by various means. This work will be her own first author paper and does not belong in our manuscript. However, the work we have presented allows us to draw the conclusion that the CAD defense pathway can be upregulated by metabolites generated by bacterial species that are typical of those in the natural environment. It provides a plausible explanation for why such a pathway has evolved, even if we do not yet know the identity of the metabolites that induce reporter expression. We could remove the section if the reviewer and editor would like, but feel that the story is stronger with it left intact.

Reviewer #1-Comment #5: The experiments with lysosomal acidity using cDCFDA should be supported by another assay (may refer https://www.biophysics-reports.org/en/article/doi/10.52601/bpr.2021.200047 or similar studies).

Authors' Response: We addressed this query by doing as the reviewer suggested. The PhsNUC-1::pHTomato reporter that the reviewer referred to (DOI: 10.52601/bpr.2021.200047) is reported to report on the pH of the lysosome, due to the pH sensitivity of the Tsien-generated pHTomato reporter that is fused to the NUC-1 protein, which localizes to lumen on the lysosome. The expression of the reporter in *C. elegans* is driven by a heat shock promoter 16.2, which drives expression in all cells (except the germline because of technical reasons) upon heat shock. According to the 2012 Li and Tsien paper that describes pHTomato, the reporter exhibits little fluorescence at pH 6 or less, moderate fluorescence at pHs between 6.5 and 8.5, and high fluorescence at higher pHs.

We were a little concerned about this reporter system because all reports of its use in *C. elegans* focus on lysosomes in the hypodermis and not the cells of the alimentary track where the CAD defense system is expressed, so it was unclear how well it would report the acidity of gut granules in the intestine, which is the focus of our previous analysis. Also note that the alimentary track is where the CADs would likely accumulate greatest and where the worm would mount the strongest response (because this is where the CAD defense components are expressed).

To do this experiment, we generated a synchronized population of XW19180 L1 reporter animals by first raising gravid XW19180 adults, which were then bleached to isolate embryos, and then subsequently incubated in M9 buffer for 24 hours. The synchronized L1 larvae were transferred into liquid Nematode Growth Medium (NGM) containing *Escherichia coli* HB101 and cultured at 20 °C. Upon reaching the L4 stage, animals expressing PhsNUC-1::pHTomato were subjected to a single heat shock treatment at 33 °C for 30 minutes as previously described (doi: 10.52601/bpr.2021.200047). Following heat shock, drug treatments were initiated by adding drugs to the liquid culture at the indicated concentrations, and the animals were incubated at 20°C for 24 in the indicated drug treatment groups.

Following the 24-hour incubation, adults (one day post-L4) were immobilized as previously described. For each treatment group, Z-stacks of five worms were captured across three independent biological replicates, and the resulting Z-series were collapsed using a **sum of slices** tool in ImageJ/Fiji software. To quantify the average pHTomato fluorescence intensity per lysosome, individual worms were isolated on the maximum intensity projections. Lysosomal puncta boundaries were segmented using an Otsu Dark threshold and segmented with a watershed algorithm. A particle filter restricted data collection to objects displaying a circularity index between 0.40 and 1.00.

We tested the same compounds we used in the cDCFDA experiments (Figure 7) plus a positive control CAD (10 mM chloroquine) that has been used by others as a positive control for other types of *C. elegans* lysosome experiments (Lu *et al.* 2022; Li *et al.* 2025). We failed to see any changes in pHTomato fluorescence in the positive control or the experimentals, despite seeing obvious induction of expression of the reporter (**Figure R1**).

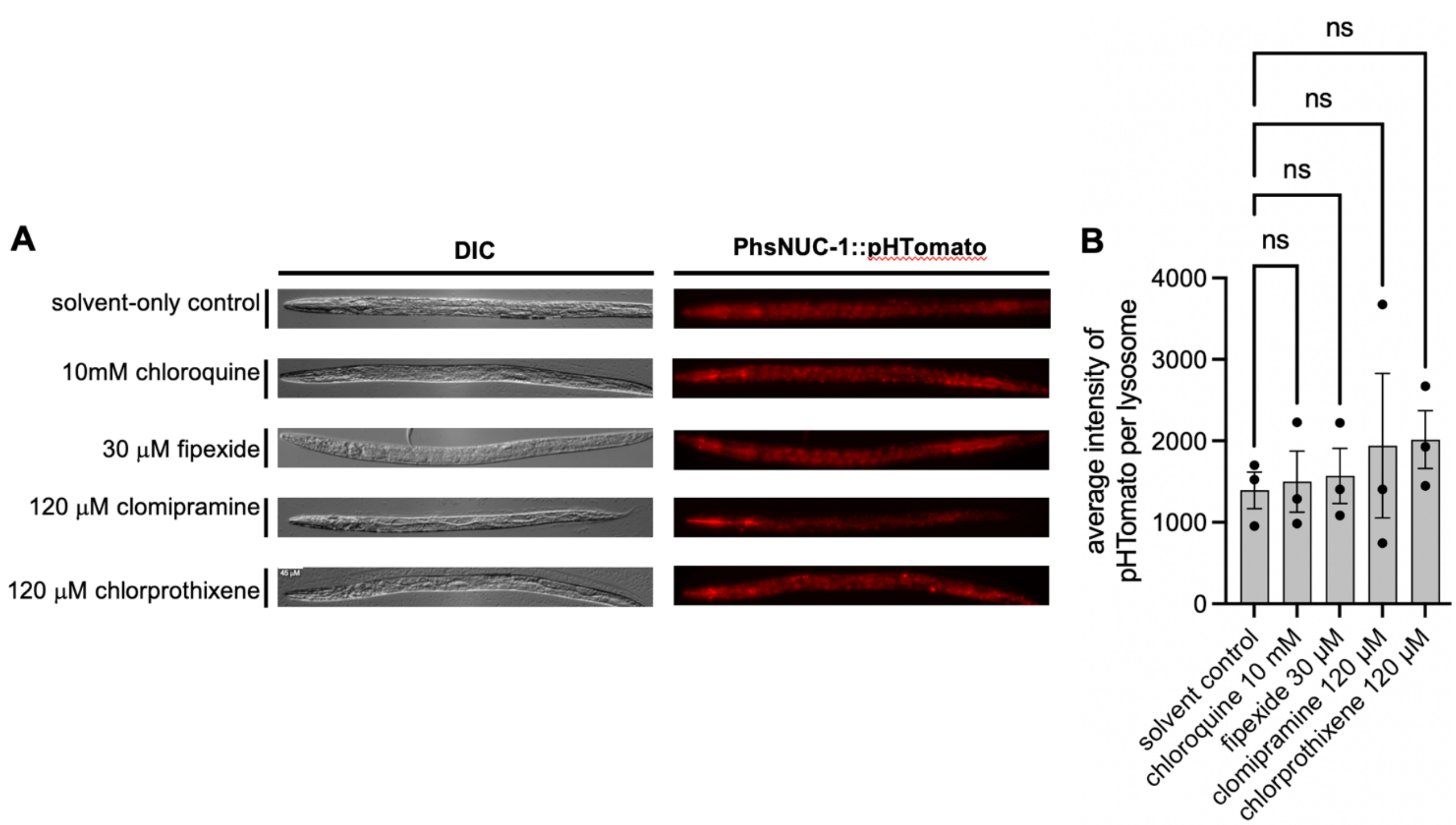

Figure R1. Testing the Utility of the PhsNUC-1::pHTomato reporter. CADs, including the positive control 10mM chloroquine, were tested for their ability to increase the intensity of the pH sensitive fluorophore pHTomato.
A. Representative images of the XW19180 PhsNUC-1::pHTomato reporter strain 24 hours after heat shock.
B. Quantification of the pHTomato fluorescent signal in the indicated conditions. Three different-day independent trials were performed and 5 worms were analyzed for each condition in each trial (N=3, n=3). One way ANOVA was used to analyze differences. ns= not significant.

We can only speculate as to why we didn't we see any effects on PhsNUC-1::pHTomato. The pHTomato assay primarily measures canonical lysosomes, such as those reported on in the hypodermis. By contrast, NHR-70 is associated with lysosome-related organelles (LROs, or gut granules) localized specifically in the intestine (TAN *et al.* 2024). As a primary site of drug absorption, the intestine experiences immediate CAD exposure, yet the pHTomato utility in the intestine has not yet been reported in the literature, raising the question of whether it is an effective tool for this tissue.

Reviewer #1-Comment #6: The authors claim, "Consistent with this interpretation, many of the gold standards that failed to induce pgp-13 expression have EC50 concentrations for PLD-induction in human cells that far exceed the concentration at which we screened in worms (30 µM) (e.g., amantadine, memantine, spectinomycin (MORELLI et al. 2006)". They need to show in the main text that higher concentrations would induce pgp-13:gfp.

Authors' Response: We tested amantadine, memantine, spectinomycin at higher concentrations in response to this request and found that none induce the expression of the *pgp-13* reporter (**Figure R2**).

We have replaced the relevant sentence to, 'It may be that some gold standards fail to induce *pgp-13* expression because of differences in pharmacokinetic access to worms compared to human cells.'

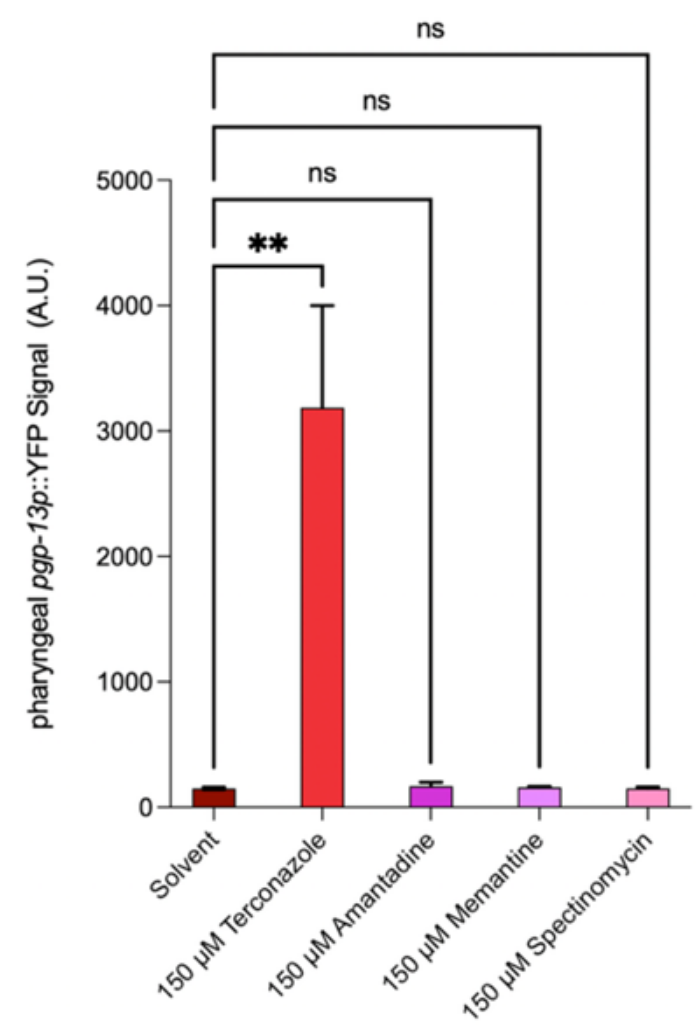

Figure R2. Gold Standard CADs Fail to Induce *pgp-13* Reporter Expression at Higher Concentrations. Compounds were tested for their ability to induce expression of the *pgp-13* reporter in living worms in the same manner as described in the manuscript. Two different-day independent trials were performed and 10 worms were analyzed for each condition in each trial. One way ANOVA was used to analyze differences. Two stars indicates p=0.006; ns= not significant.

Reviewer #1-Comment #7: In Figure 7B and C, single and double of pgp-13 and pgp-14 should be added. Are they redundant?

Authors' Response: In the original (and revised) manuscript, we provide LCMS-based evidence that PGP-13 and PGP-14 function partially redundantly to export of select CAD metabolites, but not the CADs themselves. Hence, we really didn't expect any impact of the PGP's on a phenotype that is presumably primarily dependent on the parent CAD molecules, which is why we did not present a detailed analysis of the *pgp* mutants in this analysis. However, to address this query, we tested the *pgp-13 pgp-14* double mutant and wild type controls to evaluate whether the loss the transporters modulated the gut granule staining defect phenotype; if we observed a phenotype with the double mutant, we would then do the single mutants. As expected, the *pgp-13 pgp-14* double mutant exhibits a gut granule staining pattern that is statistically indistinguishable from the wild type. We have updated Figure 7B and 7D accordingly.

Reviewer #2-Opening Remarks: This study is a very nice combination of genetic, computational, biochemical, and ecological/evolutionary approaches. It addresses an interesting question from a drug discovery and safety perspective, and contributes impactfully to the field's very early-stage understanding of xenobiotic metabolism pathways in *C. elegans*. It is quite a lot of rigorously-done work, and clearly and fairly interpreted and discussed. It is clearly written and well-organized. I am not sure I have ever had so little to say in >25 years of reviewing manuscripts.
Authors' Response: Thank you for the kind an encouraging words.

Reviewer #2-Comment #1a: Figure 1 legend: "Three independent repeats were performed (N>3); I think this should be "N = 3"?
Authors' Response: Thank you for catching this error. As reported in the original source data file tab 1 (which remains unchanged in the revision), all conditions were tested in 3 independent (different day) experiments, except for two CADs, which were teste in 6 independent experiments because of an overlap in two different students performing the experiments. We revised the sentence to, 'Either three or six independent repeats were performed (N=3 or 6)…'.

Reviewer #2-Comment #1b: …an upper ceiling of 50 animals per sample were counted…and please indicate also the lower range of animals per sample counted.
Authors' Response: We are not sure if the reviewer is a asking a question about the ceiling, but to clarify, if the well is obviously overgrown with worms, we gave it a value of 50 as is our standard published practice for manual counts. If it looked like there were fewer than 50, we manually counted how many worms were in the well (which is the lower range that the reviewer is referring to here, which could in principle be zero if that is how many worms were left in the well).

We provide additional clarity in the methods by revising a relevant sentence (new clause underlined), 'Twenty worms were deposited per well (two wells per condition and genotype) in a total volume of 50 µL, where live moving worms were quantified up to a ceiling of 50 after a six day incubation period.'

Reviewer #2-Comment #2: I recommend including a metadata file in the first tab of the Supplementary excel file.
Authors' Response: We have added a 'README' tab to the source data file that provides explanation for all subsequent tabs.

Reviewer #2-Comment #3: Page 17 "dependent" typo: "We tested whether this increase in signal was dependant on NHR-70 and NHR-107 and found that it was ($p<0.005$)"
Authors' Response: Thank you for catching this error- we have fixed it.

Reviewer #3-Opening Remarks:
This is an interesting piece of work that uses an extensive range of experimental approaches in C elegans to probe mechanisms of xenobiotic detoxification. The take home message seems to be that the nematodes can mount a defence against CADs - this is a novel finding as I understand that such a mechanism has not been identified in other organisms. The most exciting prospect would be that these systems do exist in human but are as yet undiscovered with the possibility that this work might provide a route in.
Authors' Response: We are happy that the Reviewer shares our enthusiasm for the subject matter.

Reviewer #3-Comment #1: The starting point for the study is a bit hard to follow. Is it really about PGP-14 as a model for human ABC4 deficiency? The paper writes about the pgp-14 phenotype - is this constrained to the resistance to wact-190? Or is it more extensive? If it's really about ABC4 and pgp-14 why not conduct a complementation experiment? i.e. can ABCB4 rescue pgp-14? But rather I think that their screen has provided an interesting way to reveal the interaction between PGP-13, PGP-14, upstream NHR regulation and CAD metabolism.
Authors' Response: There is a lot to unpack here; we'll take it sentence-by-sentence.

Reviewer #3-Comment #1a: The starting point for the study is a bit hard to follow.
Authors' Response: We understand that the series of chemical-genetic interactions that led to the discovery of a CAD defense system is complex. It is not by design, but simply how the series of discoveries unfolded. How we discovered the pathway is germane to our understanding of it (i.e., the CADs circumvent the need for PGP-14 because they up-regulate the PGP-13 paralog), so we think it is important to preserve this aspect of the story.

To help navigate the reader through the logic, we created Figure 1a where each successive line adds one new element to the mix. It shows how we found CADs to suppress the phenotypes associated with *pgp-14(0)*, and then, how a genetic screen revealed genetic suppressors of the ability of CADs to suppress *pgp-14(0)*. After much thought, we could find no simpler way of guiding the reader through this part of the story, but have added a cartoon as a last display item (Figure 9) to help the reader digest our findings.

Reviewer #3-Comment #1b: Is it really about PGP-14 as a model for human ABC4 deficiency?
Authors' Response: The paper's title and abstract convey the key message of the work, which is the discovery of a previously unrecognized pathway that detoxifies CADs in *C. elegans*. What led to this discovery was the search for molecules that could suppress *pgp-14(0)*. Our primary motivation for this screen was indeed to explore the use of *pgp-14(0)* mutants as a genetic model for ABCB4 deficiency to determine whether we can circumvent the loss of the PGP-14 through previously unknown routes.

We clarify this point in the revised introduction by adding the following sentence, 'We discovered this pathway serendipitously through our search for small molecules that can circumvent the disruption of a *C. elegans* homologue of a human disease gene.'

Reviewer #3-Comment #1c: The paper writes about the pgp-14 phenotype - is this constrained to the resistance to wact-190? Or is it more extensive?

Authors' Response: *pgp-14(0)* mutants are resistant to the lethality conferred by a wide variety of small molecules that form crystals in the pharynx cuticle, including wact-190. As far as we are aware, this is the only described phenotype of *pgp-14* mutants.

To clarify, we added the following new (underlined(sentence in the introduction):

> Through our previous small molecule screens (BURNS *et al.* 2015), we discovered one class of small molecules (not CADs) that kills the nematode *C. elegans* by forming crystals exclusively in the pharynx cuticle (KAMAL *et al.* 2019; KAMAL *et al.* 2022; KAMAL *et al.* 2023; KAMAL *et al.* 2024). A forward genetic screen revealed that the p-glycoprotein (PGP) pump PGP-14, which is expressed exclusively in the pharynx myoepithelium, is needed for polar lipid accumulation within the pharynx cuticle, which in turn acts as a sink for hydrophobic small molecules that form lethal crystals (KAMAL *et al.* 2019; KAMAL *et al.* 2023). Loss-of-function of *pgp-14* confers resistance to the lethality associated with crystal-forming compounds.

Reviewer #3-Comment #1d: If it's really about ABC4 and pgp-14 why not conduct a complementation experiment? i.e. can ABCB4 rescue pgp-14?
Authors' Response: As explained above, the paper's title and abstract convey the key message of the work, which is the discovery of a previously unrecognized pathway that detoxifies CADs in *C. elegans*. Earlier in the project, we did indeed try to rescue *pgp-14(0)* mutants with transgenic human ABCB4, but we did not see re-sensitization of the transgenic mutants to crystal formation. There could be many reasons for this (improper folding, localization etc etc) and the experiments were not pursued further.

Reviewer #3-Comment #1e: But rather I think that their screen has provided an interesting way to reveal the interaction between PGP-13, PGP-14, upstream NHR regulation and CAD metabolism.
Authors' Response: Precisely.

Reviewer #3-Comment #2a: If I understand correctly, the authors propose that in the pgp-14 ko CADS sensitize the worm to wact-190 because they upregulate pgp-13, which can then perform the same function in terms of promoting crystal formation.
Authors' Response: Correct.

Reviewer #3-Comment #2b: Furthermore, they reveal upstream regulation of this phenomenon. They then show that pgp-13 ko, like pgp-14 ko, can shift the dose-response for CAD lethality.
Authors' Response: Figure 7a shows that the *pgp-13(0); pgp-14(0)* double mutant is more sensitive to the lethal effects of most CADs tested than either single mutant alone, demonstrating that the two gene products act at least partially redundantly. This finding is consistent with our metabolic analyses in Figure 5b.

Reviewer #3-Comment #2c: I would like to suggest the authors rewrite some of the framing of this work to make it more 'digestible'. A cartoon would really help with the message.
Authors' Response: The series of experiments that led us to the CAD defense pathway, while logical, is admittedly complex. We did our best to walk the reader through the logic and show

the steps we took to get to the CAD defense pathway, which is illustrated in step-wise fashion in Figure 1a. In the original submission, we also provided a basic model for the relationship between the genes we uncovered and the CADs in Figure 2B. However, we welcome the suggestion by the reviewer to further expand on the model. We therefore added a new 'cartoon' model as Figure 9. We hope this provides additional clarity.

Reviewer #3-Comment #3: Why no further comment on tat-3?
Authors' Response: The short answer is that we did not want to open up a set of unresolved issues with *tat-3*; its role in the pathway deserves more investigation beyond the scope of this manuscript. Of course, it is/was proper to disclose all of the results of our forward genetic screen, which yielded *tat-3* mutants and other genes, but unlike *pgp-13*, the *cyp-35b* family members, and *cror-1*, *tat-3* is not transcriptionally up-regulated in response to CADs as measured by both qRT-PCR and a transcriptional reporter that we built (see **Figure R3**).

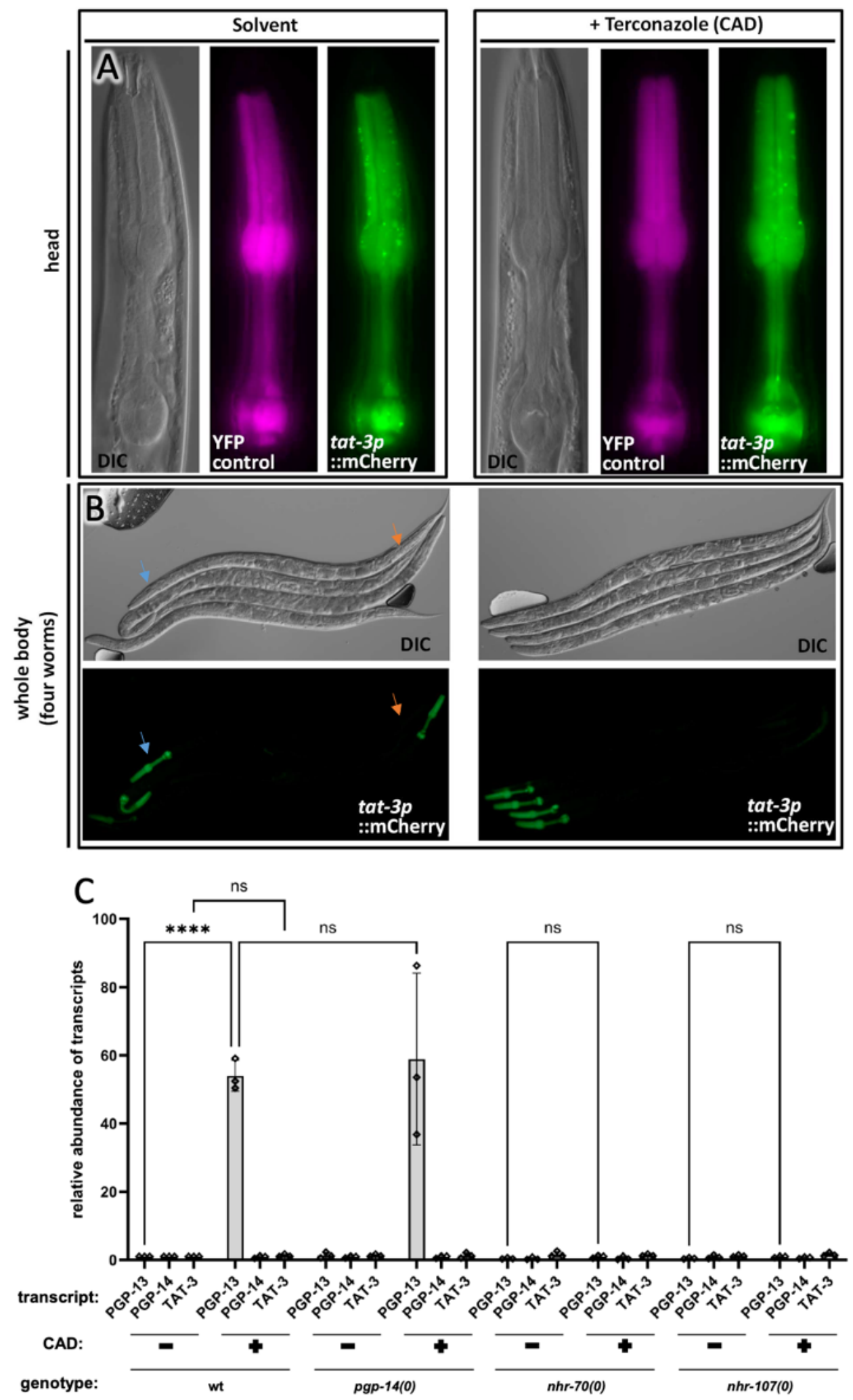

Figure R3. The CAD Terconazole Fails to Induce *tat-3* Expression.
A. An image of an RP3494 *trEx726* [*tat-3p::mCherry + myo-2p::YFP*] animal given solvent control on the left, where the expression of *tat-3* is comparable to the animals on the right exposed to 30 µM of the CAD terconazole for 24 hours. **B.** Whole animal shots of worm from the same experiment as described in A. Blue arrows indicates the anterior (head) of a single worm; orange indicates the posterior (tail) of the same worm. **C.** qRT-PCR showing that *tat-3* is not up-regulated in expression in response to growth in 30µM terconazole for 96 hours. **** = p<0.001.

We can speculate on the role of TAT-3 in the pathway based on what is known about the protein. TAT-3 is one of six subfamily IV P-type ATP-ase (P4 ATPase) expressed by the worm (Darland-Ransom et al., 2008). P4 ATPases function as lipid flippases that maintain the asymmetric distribution of phospholipids within the plasma membrane (Darland-Ransom et al., 2008; Lyssenko et al., 2008). In a typical plasma membrane, phosphatidylserine and phosphatidylethanolamine are concentrated in the inner, cytosolic leaflet, whereas phosphatidylcholine and sphingomyelin are mostly in the outer leaflet (Fujimoto and Parmryd, 2017). P4-ATPases maintain this asymmetry by typically transporting phosphatidylserine and phosphatidylethanolamine from the outer to the inner leaflet (Lopez-Marques et al., 2014; Andersen et al., 2016). Our work (**Figure R3A**) and that of others (LYSSENKO *et al.* 2008) shows that *tat-3* is expressed in the pharyngeal muscle, marginal cells, and the buccal epithelium.

tat-3 loss-of-function mutants phenocopy *pgp-13* loss-of-function with respect to the suppression of CADs' ability to rescue *pgp-*

14(0)'s insensitivity to wact-190 crystal-inducing death (a lot to process, we know). We therefore speculate that TAT-3 may be required to maintain a proper lipid composition in the plasma membrane where PGP-14 resides in the pharynx, without which PGP-14 cannot function optimally. This is analogous to the need of specific membrane architecture for the proper functioning of other ABCB transporters like PGP-14's homologue ABCB4 (ZHAO *et al.* 2015). Models involving transporter trafficking can also be contemplated (Underwood, Ryan and Somerville, 2017; Milne, Edeen and Fay, 2025). Regardless, because there is no evidence of TAT-3 expression in the intestine, it is unlikely that it plays any important role in CAD defence and its inclusion in this current manuscript would be a digression.

Reviewer #3-Comment #4a: Could the authors comment more explicitly on the baseline effect of the CADs in the study on wild-type?
Authors' Response: As seen in Figure 7A, the CADs have variable effects on the growth rate of the population of wild type animals compared to no-CAD controls (i.e., the zero micromolar concentration of each drug in wild type). Beyond lethality, we have not observed any other obvious 'Brenner' type of plate defeats in the worms behaviour or development. We added a comment in the relevant results section in the revised manuscript as follows, 'In the wild type, the CADs have different lethal potencies, depending on their structure (Figure 7A).'

Reviewer #3-Comment #4b: And how the dosing was chosen for the pgp-14 ko screen in this regard?
Authors' Response: We are uncertain as to which screen involving *pgp-14(0)* the Reviewer is referring to. First, we'll address the query based on the Reviewer asking about the screen of small molecules for those that can rescue the *pgp-14(0)* phenotype (i.e., the screen for rescue of *pgp-14(0)*'s insensitivity to wact-190 crystal-inducing death) (i.e., line 4 in Figure 1A) that yielded the CAD molecules. In this screen, we used 15 micromolar wact-190 in the background of *pgp-14(0)*. 15 micromolar wact-190 is about 10 fold higher concentration than the LD100 to kill wild type (Kamal et al., 2019, 2022, 2024). We went with a high concentration of wact-190 to ensure that any re-sensitization of *pgp-14(0)* would be readily detected. We screened the Spectrum small molecules at a concentration of 30 micromolar, which was chosen based on two decades of our experience in doing small molecule screens with *C. elegans* and the doses at which many molecules accumulate in the worm but do not necessarily kill it (e.g., Burns et al., 2005, 2010, 2015). This concentration is also in line with that used by others (see (O'REILLY *et al.* 2014)). This screen yielded the CAD molecules.

If the Reviewer is asking about the dose of terconazole that was used in the forward genetic screen for gene products that mediate CAD sensitivity (i.e., line 6 in Figure 1, which yielded *pgp-13* mutants etc), we chose 30 micromolar of terconazole because it was far below (~five fold less than) the LD100 in wild type animals. Evidently, mutants lacking the NHR-70-PGP-13 CAD defense pathway can still tolerate that concentration of terconazole, otherwise, we would would not have been able to isolate the CAD-insensitive mutants.

Reviewer #3-Comment #5: Fig 7. How are the worms identified as dead? Is it possible they are just immobilised? What was the stage of the worm? Adult? What was the exposure time?
Authors' Response: We provide answers to these questions in the methods section related to Figure 7. In brief, the worms used in the assay associated with Figure 7 were synchronized L1s, and the exposure time to the drug or solvent was 3 days. Regarding their viability, worms that remained mobile after this period were considered viable, whereas immobile worms that developmentally arrested at the L1/L2 stage were considered dead. While it is possible for compounds to induce paralysis, the combination of complete immobility and prolonged developmental arrest after a 3-day exposure serves as our metric for lethality similar to all of our other published works.

Reviewer #3-Comment #6: Typos; page 9, 17 and possibly elsewhere- 'dependant' should be 'dependent'
Authors' Response: We thank the reviewer for bringing this to our attention. We have fixed the error.

Reviewer #3-Comment #7: Fig 1 legend D. should this really be 30mM?
Authors' Response: Nice catch. We have fixed the error.

Reviewer #3-Comment #8: P 23 typo 'Hence, we endeavored To'
Authors' Response: We have fixed the error.

Literature Cited

Arda, H. E., S. Taubert, L. T. MacNeil, C. C. Conine, B. Tsuda *et al.*, 2010 Functional modularity of nuclear hormone receptors in a Caenorhabditis elegans metabolic gene regulatory network. Mol Syst Biol 6**:** 367.

Burns, A. R., G. M. Luciani, G. Musso, R. Bagg, M. Yeo *et al.*, 2015 Caenorhabditis elegans is a useful model for anthelmintic discovery. Nat Commun 6**:** 7485.

Kamal, M., J. Knox, R. I. Horne, O. S. Tiwari, A. R. Burns *et al.*, 2024 A Rapid in vivo Pipeline to Identify Small Molecule Inhibitors of Amyloid Aggregation. Nature Communications Accepted.

Kamal, M., H. Moshiri, L. Magomedova, D. Han, K. C. Q. Nguyen *et al.*, 2019 The marginal cells of the Caenorhabditis elegans pharynx scavenge cholesterol and other hydrophobic small molecules. Nat Commun 10**:** 3938.

Kamal, M., L. Tokmakjian, J. Knox, D. Han, H. Moshiri *et al.*, 2023 PGP-14 establishes a polar lipid permeability barrier within the C. elegans pharyngeal cuticle. PLoS Genet 19**:** e1011008.

Kamal, M., L. Tokmakjian, J. Knox, P. Mastrangelo, J. Ji *et al.*, 2022 A spatiotemporal reconstruction of the C. elegans pharyngeal cuticle reveals a structure rich in phase-separating proteins. Elife 11.

Li, T. Y., A. W. Gao, R. Yang, Y. Sun, Y. Lei *et al.*, 2025 A lysosomal surveillance response to stress extends healthspan. Nat Cell Biol 27**:** 1083-1097.

Liu, J., A. Malekoltojari, A. Asokakumar, V. Chow, L. Li *et al.*, 2024 Diindoles produced from commensal microbiota metabolites function as endogenous CAR/Nr1i3 ligands. Nat Commun 15**:** 2563.

Lu, R., J. Chen, F. Wang, L. Wang, J. Liu *et al.*, 2022 Lysosome Inhibition Reduces Basal and Nutrient-Induced Fat Accumulation in Caenorhabditis elegans. Mol Cells 45**:** 649-659.

Lyssenko, N. N., Y. Miteva, S. Gilroy, W. Hanna-Rose and R. A. Schlegel, 2008 An unexpectedly high degree of specialization and a widespread involvement in sterol metabolism among the C. elegans putative aminophospholipid translocases. BMC Dev Biol 8**:** 96.

Motta, S., L. Callea, S. Giani Tagliabue and L. Bonati, 2018 Exploring the PXR ligand binding mechanism with advanced Molecular Dynamics methods. Sci Rep 8**:** 16207.

O'Reilly, L. P., C. J. Luke, D. H. Perlmutter, G. A. Silverman and S. C. Pak, 2014 C. elegans in high-throughput drug discovery. Adv Drug Deliv Rev 69-70**:** 247-253.

Tan, C. H., T. Y. Wang, H. Park, B. Lomenick, T. F. Chou *et al.*, 2024 Single-tissue proteomics in Caenorhabditis elegans reveals proteins resident in intestinal lysosome-related organelles. Proc Natl Acad Sci U S A 121**:** e2322588121.

Zhao, Y., M. Ishigami, K. Nagao, K. Hanada, N. Kono *et al.*, 2015 ABCB4 exports phosphatidylcholine in a sphingomyelin-dependent manner. J Lipid Res 56**:** 644-652.

APPENDIX A: NHR70 and NHR107 Are Not Activated by the Tested Compounds in Luciferase Reporter Assays on Human Cell Line

To determine if NHR70 and NHR107, could be activated by CADs, we established luciferase reporter gene assays. Human Pregnane X Receptor (hPXR) was served as a benchmark. HepG2 cells expressing either NHR70 or NHR107 were treated with a panel of compounds known to interact with various nuclear receptors, alongside a potent hPXR agonist, SR12813. The robust activation of hPXR by both SR12813 and Nicardipine confirmed the assay's function. However, neither NHR70 nor NHR107 exhibited notable induction in luciferase activity in response to any of the tested drugs, compared to the strong induction observed for hPXR. The maximum activation for both NHR70 and NHR107 was less than 2-fold, while hPXR reached approximately 100-fold induction. To confirm that the lack of activation was not due to poor receptor expression, we performed Western blotting. We demonstrated protein expression of both NHR70 and NHR107 at their expected molecular weights in the transfected cells.

Cell culture reporter assays. HepG2 cells were maintained in RPMI 1640 media (Gibco, 11870593) containing 10% fetal bovine serum (FBS) and 1% Penicillin/Streptomycin (P/S) at 37°C in a humidified atmosphere of 5% CO_2. For transfection assays, HepG2 cells were cultured into a clear bottom white 96-well plate in RPMI 1640 media (Gibco, 11870593) containing 10% FBS. After 24 hrs incubation, cells were transfected with the plasmid DNA using Lipomaster 3000 (Vazyme, TL301-01). The plasmid DNA contained 50 ng of UAS-luciferase reporter, 15 ng of GAL4-NHR70, or GAL4-NHR107, or GAL4-hPXR, 20 ng of the *Renilla reniformis* luciferase transfection control plasmid, and 65 ng of pGEM filler vector. Six hours after transfection, cells were treated with vehicle (DMSO) or various concentrations of compounds. The following day (16 hrs after ligand addition), luminescence activity in the cell lysate was measured using Dual-Luciferase Reporter Assay (Promega, Madison, WI, USA).

Western blotting. Total protein was extracted using Passive lysis buffer (E1941, Promega) containing protease inhibitor cocktail (Thermo Fisher Scientific, 78430) for 30 min on ice. Protein was quantified by BCA, then 2 × loading buffer was added, incubated at 95 °C for 5 min and separated on 12% SDS gels. Samples were wet-transferred onto nitrocellulose membrane (GE Healthcare, Chicago, Illinois, USA) at 200 mA for 60 min. Blocking was performed for 15 min at room temperature in blocking solution (5% skim milk in PBS with 0.05% Tween-20 (PBST)), incubations with the primary and secondary antibodies were performed as suggested by the manufacturer. Amersham ECL Western Blotting Detection Reagent (Cytiva, RPN2109) was used for signal detection. Blots were exposed to an X-ray film (Thermo Fisher Scientific, 34091) for 30–60 seconds. Uncropped blots are available in the provided Source Data file.

Monoclonal Anti-FLAG® M2 antibody (F3165, 1:1000) was purchased from Sigma.
Polyclonal β-Actin antibody (4967, 1:1000) was purchased from Cell Signaling Technology.

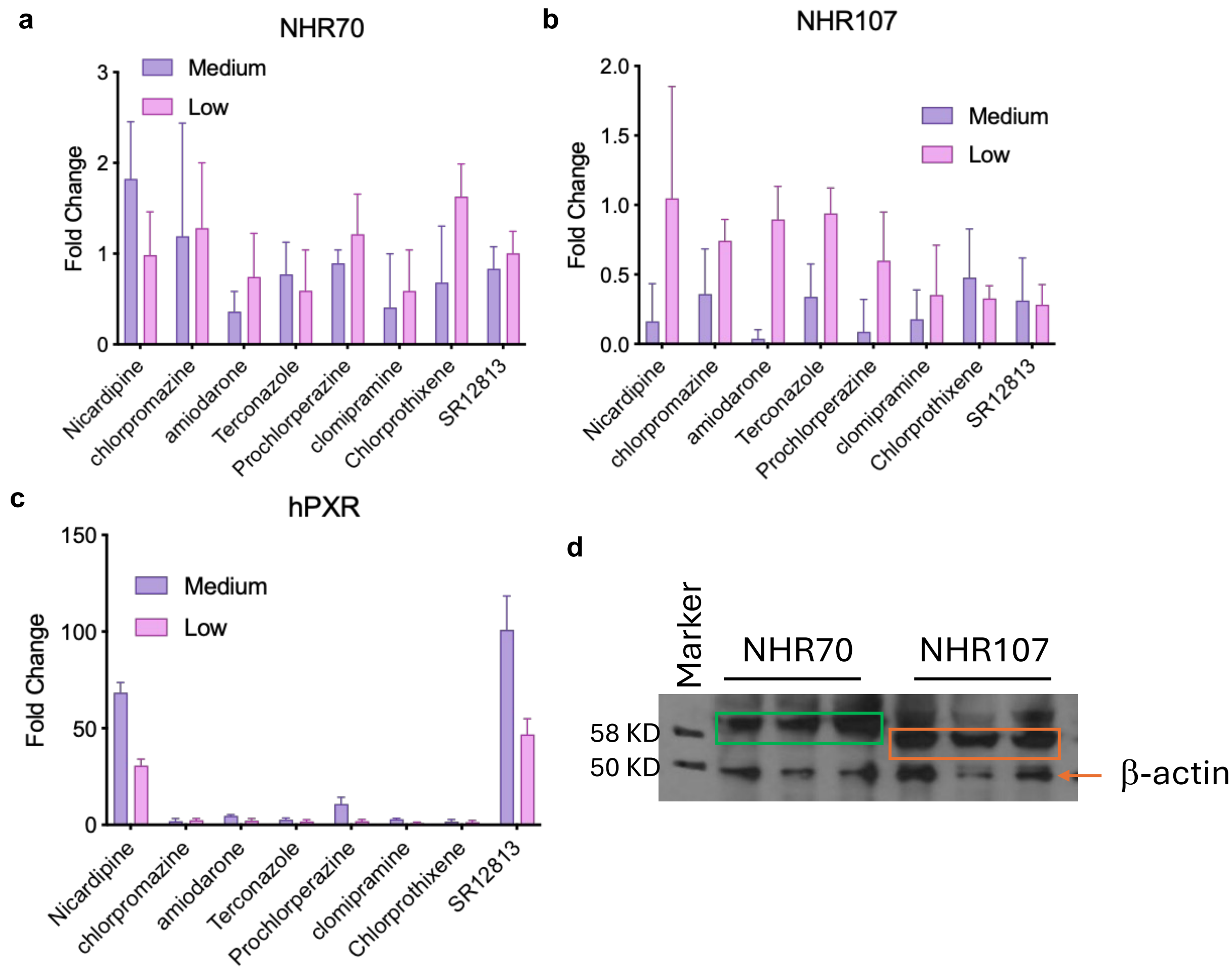

Appendix A Figure 1. Screening of CADs for NHR70 and NHR107 activation.
(a–c) HepG2 transfected with UAS-luciferase reporter constructs and expression plasmids for (a) Gal4-NHR70, (b) Gal4-NHR107, or (c) Gal4-human PXR were treated with medium (10 μM for CADs, 1 μM for SR12813, purple bars) and low (1 μM for CADs, 0.1 μM for SR12813, pink bars) concentrations of various indicated drugs (e.g., Nicardipine, chlorpromazine). Data are presented as fold change in normalized luciferase activity relative to vehicle control. Data are expressed as means ± SD (n = 3 technical replicates). (d) Western blot showing the protein expression of NHR70 and NHR107 in the transfected cell lines. Cell lysates from NHR70 and NHR107-expressing cells were probed with anti-FLAG antibody. Green and orange boxes highlight the NHR70 and NHR107 protein bands, respectively, at 56.3 kDa (predicted) and 54.5 kDa (predicted). β-actin is used as a loading control, with molecular weight markers of 58 kD and 50 kD indicated.

APPENDIX B: Virtual Screening of CADs Against NHR-70 and NHR-107: Analysis Report

Two *C. elegans* nuclear receptors (NHR-70, NHR-107) were each co-folded with four cationic amphiphilic drugs (CADs) terconazole, amiodarone, chlorprothixene, and clomipramine in both charged and uncharged protonation states using AlphaFold3 (AF3) (https://doi.org/10.1038/s41586-024-07487-w) and Boltz-2 (https://doi.org/10.1101/2025.06.14.659707).

In these docking experiments, we also included the human PXR nuclear receptor, which is well characterized to bind the PXR agonists SR12813 and hyperforin as positive controls. Of note, there is some weak evidence in the literature that PXR mediates a response to some CADs, but direct physical interaction remains an open question. We also included the human VDR nuclear receptor as a negative control because it has not been reported to interact with the SR12813 and hyperforin ligands.

For all predictions, the Ligand Binding Domain (LBD) sequence was used in addition to the N-terminal linker region as a buffer region. The top-ranked structure was selected from each run, using the ranking score for AF3 and the iPTM score for Boltz-2. GNINA v1.3.2 (https://doi.org/10.1186/s13321-025-00973-x, https://doi.org/10.1186/s13321-021-00522-2) was then used to re-dock each ligand into its predicted receptor structure, generating Vina and CNN-based affinity and pose scores across all receptor–ligand–protonation combinations. GNINA CNN affinity approximates $-\log_{10}(Kd)$, where a score of 5 corresponds to ~10 µM, 6 to ~1 µM, 7 to ~100 nM, and 8 to ~10 nM predicted binding affinity. Each unit increase reflects a 10-fold improvement. CNN pose score reflects the predicted probability that a docked pose is within 2 Å RMSD of the true binding mode, where higher scores indicate greater geometric confidence. We interpret these scores as relative rankings across receptors and ligands rather than absolute affinity predictions and compare them within each tool to assess whether NHR-70 and NHR-107 score similarly to PXR or VDR.

To assess the validity of the structures, RMSD was calculated between AF3 and Boltz-2 predictions aligned on the LBD Cα atoms only, excluding the flexible N-terminal linker. Both tools accurately reproduced the PXR (2O9I) and VDR (1DB1) crystal structures at sub-angstrom to low-angstrom precision (AF3: 0.76–0.79 Å for PXR, 1.18–1.39 Å for VDR; Boltz-2: 0.83–0.94 Å for PXR, 0.48–0.59 Å for VDR), validating the predicted folds (Figure 1A). Inter-tool LBD agreement was strong across all receptors, with all combinations falling at or below the 2 Å threshold (Figure 1B; mean LBD Cα RMSD: NHR-70 0.80 Å, NHR-107 1.65 Å, PXR 1.14 Å, VDR 0.96 Å). For PXR and VDR, inter-tool RMSD was computed over residues covered by the respective crystal structure (Figure 1C) to exclude flexible C-terminal extensions not resolved in the PDB.

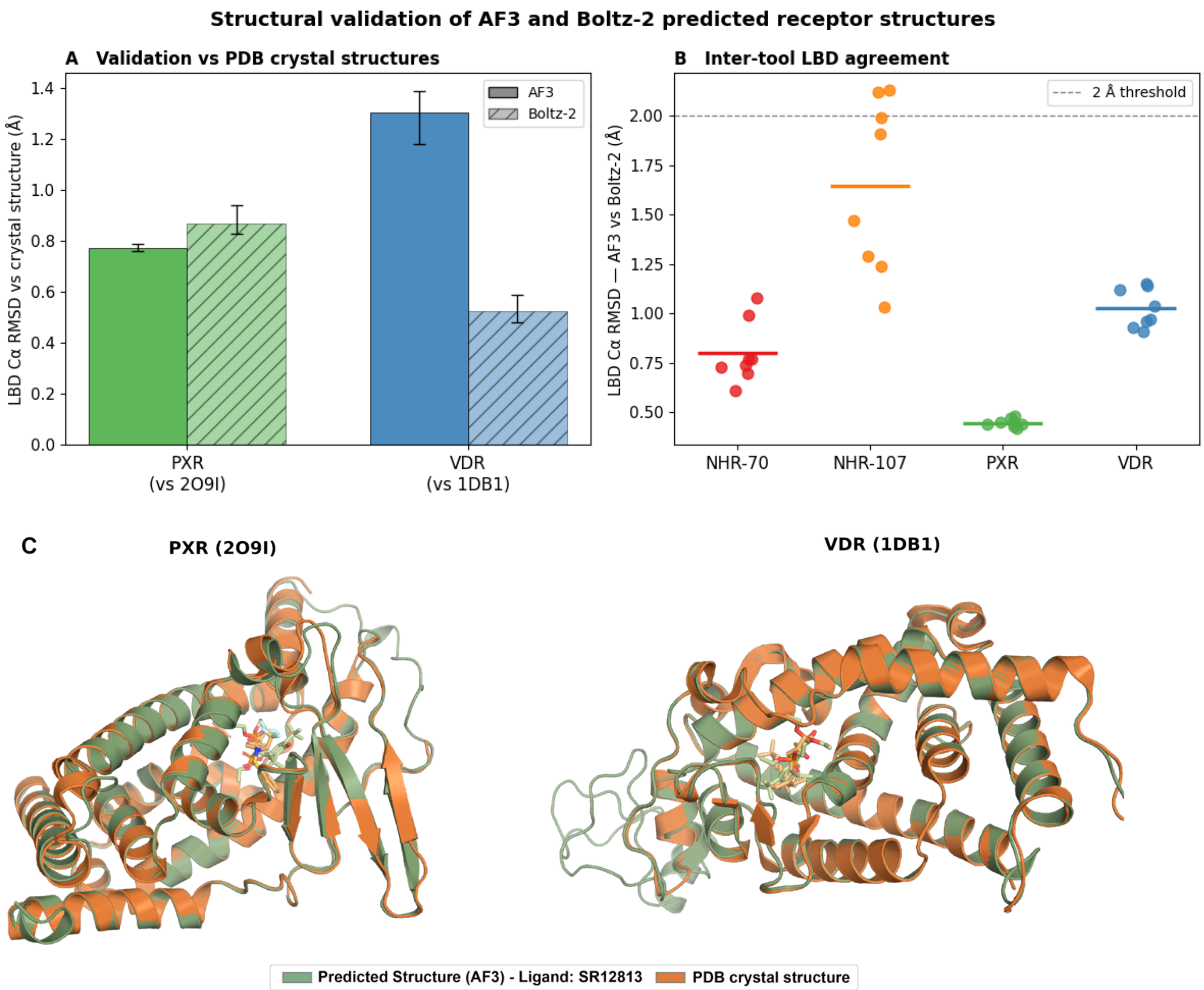

Appendix B Figure1. Structural validation of AF3 and Boltz-2 predicted receptor structures. (A) Mean LBD Cα RMSD of AF3 (solid) and Boltz-2 (hatched) predictions versus PDB crystal structures for PXR (2O9I) and VDR (1DB1). Error bars show the min–max range across eight ligand–protonation combinations. (B) Inter-tool LBD Cα RMSD between AF3 and Boltz-2 for all four receptors (n = 8 per receptor); horizontal bars indicate the mean. (C) Structural overlay of AF3-predicted (green) and PDB crystal (orange) LBDs for PXR (left) and VDR (right), with SR12813 shown as sticks.

Before conducting the computational docking experiments, we had three benchmarks to gauge whether the docking experiments were yielding useful predictions.

First, ***if the CADs physically interact with NHR-70 and NHR-107 AND the computational docking experiments were predictive, we expected that the charged versions of CADs to yield greater affinity for the C. elegans receptors relative to the uncharged versions*** because the CADs would be largely charged within the cell. In AF3-based docking, the uncharged forms scored nearly always higher (i.e. a predicted greater affinity) than charged forms with NHR-70 and NHR-107 (Figures 2A & B). Similar results are found using Boltz-2 (Figures 3A & 3B). ***Hence, the computational predictions failed to meet this benchmark.***

Second, experiments using *nhr-70(0)* and *nhr-107(0)* mutants indicated that terconazole, chlorprothixene, and clomipramine depend on both NHR-70 and NHR-107 to drive the CAD defense response. By contrast, amiodarone is much more dependent on NHR-70 than on NHR-107. Hence, ***if the CADs physically interact with NHR-70 and NHR-107 AND the computational docking experiments were insightful, we would expect that amiodarone to have a higher predicted affinity for the NHR-70 ligand compared to NHR-107.*** In the charged state, AF3 CNN affinity (Figure 2A) marginally favored NHR-70 (6.66 vs 6.51) and pose score (Figure 2D) clearly favored NHR-70 (0.75 vs 0.62). In the uncharged state, the trends reversed (Figure 2B,E).With Boltz-2, there was little difference with in predicted affinity with charged amiodarone Figure 3A), and the pose score favoured NHR-107, not NHR-70 (Figure 3D), with similar results for uncharged amiodarone (Figures 3B & 3E). ***Hence, this benchmark is not clearly met.***

Finally, ***if the computational docking experiments were indeed predictive, we expected PXR to show higher predicted affinity for SR12813 and hyperforin than VDR***. Here, the trends for affinity are all consistent for the positive control ligands (Figure 2C and Figure 3C), and the pose scores are largely in agreement with expectations (Figure 2F and Figure 3F). ***Hence, the computational predictions are in reasonable agreement with expectations with the positive controls,*** *but the magnitude of the differences between the positive control pairs and the negative control pairs is expected to be larger.*

Taken together, the results do not allow a definitive conclusion about whether the CADS are ligands for NHR-70 and NHR-107.

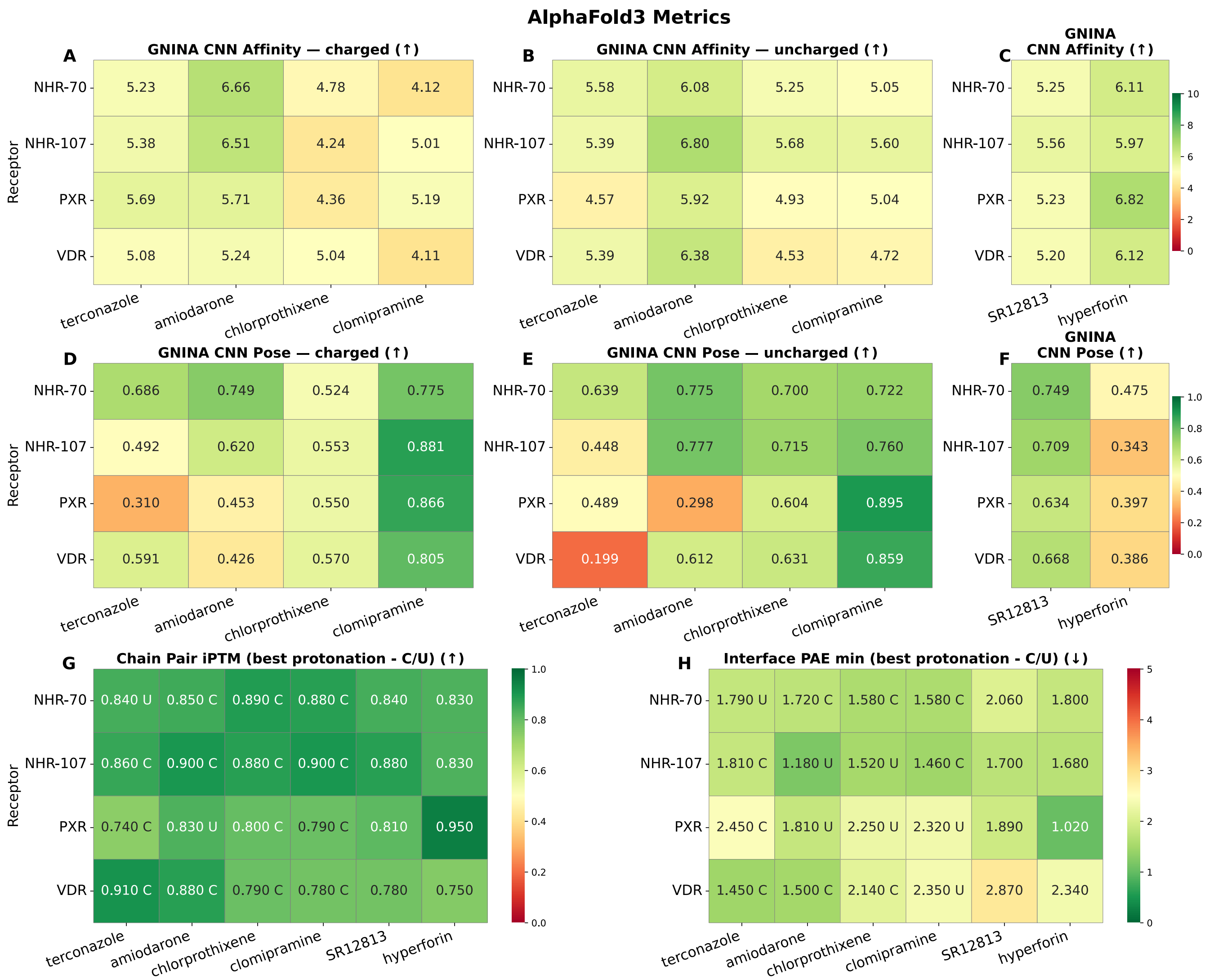

Appendix B Figure 2. AlphaFold3 co-folding and GNINA re-docking scores across all receptor–ligand combinations. Panels A–C show GNINA CNN affinity (↑ higher = stronger predicted binder) and panels D–F show CNN pose score (↑ higher = more realistic binding geometry), each split into charged (A, D), uncharged (B, E), and PXR control ligands SR12813 and hyperforin (C, F). Note that protonation states only differ for the four CADs; SR12813 and hyperforin were modelled in a single neutral form. Panels G–H show AF3 internal confidence metrics: chain pair iPTM (G; ↑ higher = more confident ligand placement) and interface PAE min (H; ↓ lower = less positional uncertainty between receptor and ligand chains), each displaying the best-scoring protonation state per cell (C = charged, U = uncharged).

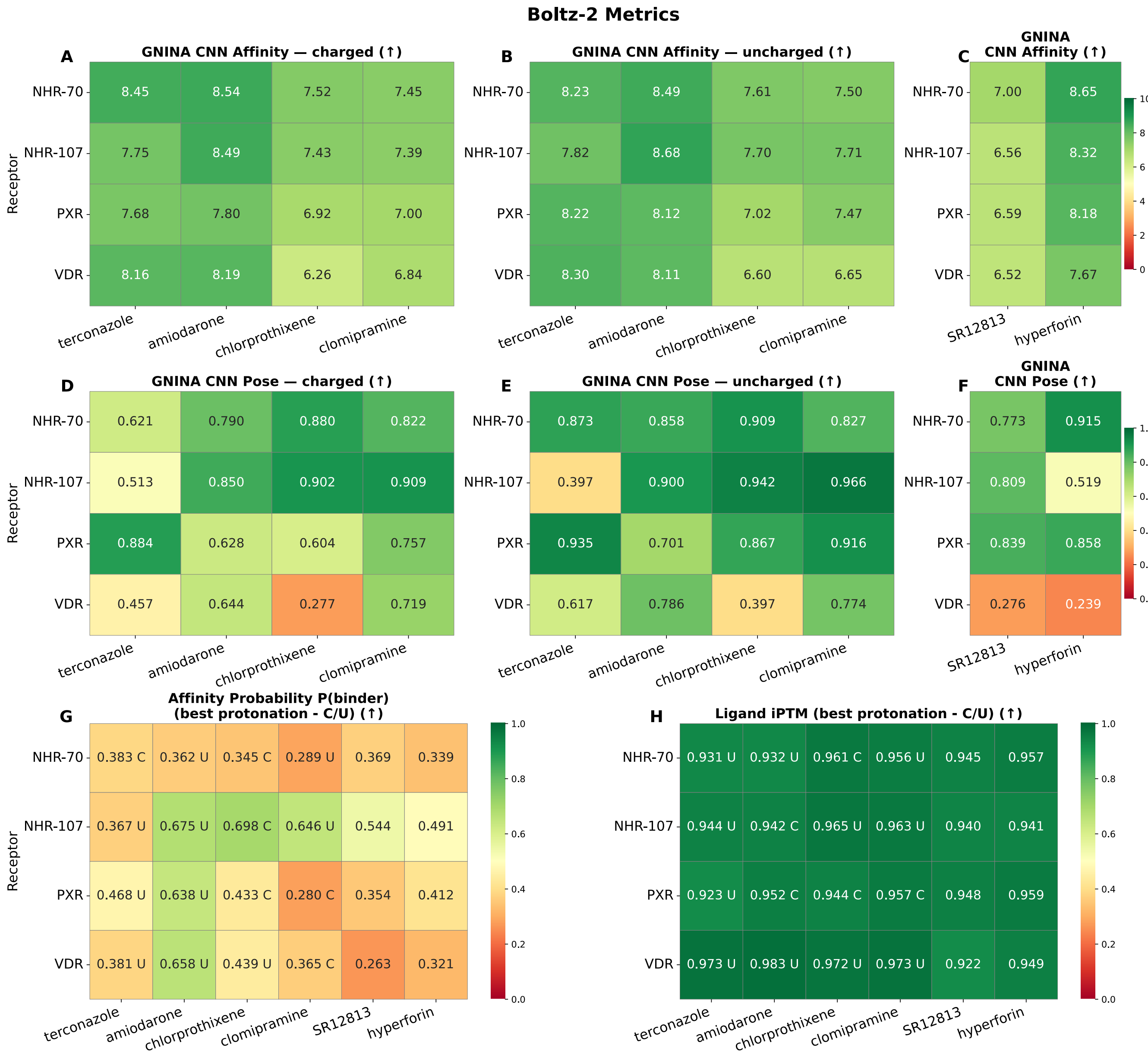

Appendix B Figure 3. Boltz-2 co-folding and GNINA re-docking scores across all receptor–ligand combinations. Panels A–C show GNINA CNN affinity (↑ higher = stronger predicted binder) and panels D–F show CNN pose score (↑ higher = more realistic binding geometry), each split into charged (A, D), uncharged (B, E), and PXR control ligands SR12813 and hyperforin (C, F). Note that protonation states only differ for the four CADs; SR12813 and hyperforin were modelled in a single neutral form. Panels G–H show AF3 internal confidence metrics: chain pair iPTM (G; ↑ higher = more confident ligand placement) and interface PAE min (H; ↓ lower = less positional uncertainty between receptor and ligand chains), each displaying the best-scoring protonation state per cell (C = charged, U = uncharged).
