# Supplementary material for: The nematode Caenorhabditis elegans has a cationic amphiphilic drug (CAD) defense system
Source: PLoS Biol. 2026 Sep 21;24(9):e3004006. doi: 10.1371/journal.pbio.3004006 (PMC13619054; doi:10.1371/journal.pbio.3004006)
Supplement: Supplementary file 3 [file pbio.3004006.s018.pdf]

Editors Comments:

Preamble: Thank you for your patience while we considered your revised manuscript entitled "A Cationic Amphiphilic Drug (CAD) Defense System in the Nematode Caenorhabditis elegans" for publication as a Research Article at PLOS Biology. This revised version of your manuscript has been evaluated by the PLOS Biology editors, the Academic Editor and one of the original reviewers.

Editor Comment 1: Based on the comments (attached below), we are likely to accept this manuscript for publication, provided you satisfactorily address the remaining points raised by Reviewer 1.
Our response: We have addressed each of their points below as requested by them. We hope this is satisfactory.

Editor Comment 2: After consulting with the Academic Editor, we agree that all the negative results should be reported in the manuscript.
Our response: We have implemented this suggestion. We have added the supplementary data to the supplementary figures as requested by Reviewer #1.

Editor Comment 3: The statement that "CADs may directly interact with the nuclear receptors" is fine.
Our response: OK- we have left that as is.

Editor Comment 4a: In point 4, you could replace "at least as good as" with "a good predictor" as 78% vs 73% is in the margin of error.
Our response: We have implemented this suggestion. We have changed the original sentence, 'Hence, our *in vivo* CAD defence reporter is at least as good as a computational predictor of CAD-induced pathologies in humans and provides a facile model for testing predictions.' to, 'Hence, our *in vivo* CAD defence reporter is a good *in vivo* predictor of CAD-induced pathologies in humans.'

Editor Comment 4b: You could also say that "C. elegans may increase its survival rate in the presence of CAD-producing microbes."
Our response: We have implemented this suggestion. We have changed the relevant sentence from, 'These observations suggest that a CAD defense pathway may have evolved in *C. elegans* to increases its survival rate in the presence of CAD-producing microbes.', to, 'Together, these observations suggest that a CAD defense pathway may help *C. elegans* survive in the presence of CAD-producing microbes.'

Editor Comment 5: In addition, we would like you to consider a suggestion to improve the title, "C. elegans have a cationic amphiphilic drug (CAD) defense system".
Our response: We have implemented this suggestion. Our original title was, 'A Cationic Amphiphilic Drug (CAD) Defense System in the Nematode *Caenorhabditis elegans*'. We have revised it to something similar to what you have suggested: '*Caenorhabditis elegans* has a Cationic Amphiphilic Drug (CAD) Defense System'.

Editor Comment 6: As you address these items, please take this last chance to review your reference list to ensure that it is complete and correct. If you have cited papers that have been retracted, please include the rationale for doing so in the manuscript text, or remove these references and replace them with relevant current references. Any changes to the reference list should be mentioned in the cover letter that accompanies your revised manuscript.

Our response: It is not clear whether there is a specific concern here or whether this passage included with all manuscripts at this stage? Regardless, we are not aware that any of the cited papers are retracted. Furthermore, we used AI (Perplexity) to check the list of papers for retractions and it too could also not find evidence that any of the cited papers were retracted. If there is a specific concern, please let us know and we will deal with it.

Reviewer 1's comments

Reviewer 1 Preamble: I thank the authors for the extensive new work and for a rebuttal that is candid about experiments that did not succeed. My concern is narrow: four experiments were done at my request, all returned negative results, and none appear in the manuscript.

Reviewer 1-Comment 1a: Three approaches — GAL4-LBD/luciferase in HepG2, AlphaFold3/Boltz-2 docking with PXR and VDR benchmarks, and LBD pull-downs with LC-MS, all returned negative results but are not reported in the manuscript; a reader would never know about these. "Remains an open question" no longer fairly describes what you know.
Our response: We thank the reviewer for the careful consideration of our work. With respect to the issue of whether the CADs interact directly or not with the NHRs, none of the new data presented is conclusive. For example, the negative data of the heterologous HepG2 experiments is easily explained away by the possibility that unknown *C. elegans* co-factors also need to be expressed. The computational analyses did not yield definitive results, and the LBD pull-downs were preliminary. We would feel uncomfortable stating that the CADs do not interact with the NRs and we clearly do not have evidence that the CADs directly interact with the NRs. Hence, we feel that the question remains open.

Reviewer 1-Comment 1b: Please add Appendices A and B as supplemental figures with brief text, including your caveats about absent worm-specific cofactors.
Our response: We have implemented this suggestion. We have added the display items from the appendices to the Supplemental Figures (4 and 5), added a corresponding methods section, and have added the following sentences to the appropriate results section:

> Third, we tested whether expression of ligand-binding domain fusions of NHR-70 and NHR-107 to the GAL4 DNA-binding domain could drive reporter expression in HepG2 cells in response to CADs and found that they could not (Supplemental Figure 4). It is possible that this heterologous system lacks other *C. elegans* proteins necessary drive NHR-dependent transcription. Finally, we also performed computational modeling to predict whether the CADs directly interact with each of the NHR's ligand-binding domains (Supplementary Figure 5). The positive controls behaved weaker than expected and the modeling provided no evidence of direct interaction. Hence, we consider the question of how the CADs interact with the NHRs an open question.

Reviewer 1-Comment 1c: New Figure 9 opens with "CADs may directly interact with the nuclear receptors." Please give the two arms of the model equal weight and cite the negative data in the legend.
Our response: The figure's title is neutral. The first sentence describes model 1 and the second sentence describes model 2. Furthermore, the figure clearly indicates that the interaction could be direct or indirect. It is not clear how equal weight is not given. For posterity, it should be noted that we have no preference for either model; we simply lack the data the discerns them and that a model of direct interaction, like that observed with the P450s, is the simplest model to present and is therefore presented first.

Reviewer 1-Comment 2a: pHTomato showed no change in response to any CAD or to 10 mM

chloroquine. The failed positive control indicates that the assay was uninformative for intestinal LROs; this is worth publishing, as it will spare others the effort. Please report Figure R1.
Our response: We have implemented this suggestion. We have converted R1 to Supplemental Figure 10 and added a corresponding methods section. We have also added the following sentence to the relevant results section, 'Of note, we also tested the pHTomato reporter (Li et al. 2021), but it failed to generate signal with our positive control (10 mM chloroquine) (Supplemental Figure 10).

Reviewer 1-Comment 2b: cDCFDA therefore remains the sole pathology readout, and phospholipidosis itself is still never measured. Please state in the Discussion that the relationship to PLD is inferred.
Our response: To clarify, nowhere do we assert that CADs induce PLD in *C. elegans*. However, to add clarity to this point in the manuscript, we have made three changes in the newly revised manuscript.

First, we added the underlined words to the following sentence in the abstract, 'Cationic Amphiphilic Drugs (CADs) severely disrupt lysosomal function, which leads to a cellular pathology <u>in humans</u> characterized by excess phospholipids called phospholipidosis.'

Second, we added the underlined words to the following sentence in the results section, 'Second, we compared the *pgp-13*-inducers to the Spectrum molecules predicted to be PLD-inducing CADs <u>in humans</u>.'

Third, we added the following sentence to the end of the 'The CAD Detoxification Pathway Protects Against Pathogenesis' results section, 'Whether CADs induce phospholipidosis in *C. elegans* is an open question.'

Reviewer 1-Comment 3: Amantadine, memantine and spectinomycin were tested at higher concentrations and none induced (Figure R2). The revision replaces the original claim with an uncheckable restatement of the same permeability hypothesis while the disconfirming data stay unpublished. Please include Figure R2 and state in the Results that the concentration explanation does not hold.
Our response: We have implemented this suggestion. We have included R2 as a supplemental figure and revised the relevant section as follows:

> We tested whether three of the failed gold standards would elicit *pgp-13* reporter expression at higher concentrations and found that they did not (Supplemental Figure 7). It may be that some gold standards fail to induce *pgp-13* expression because of differences in pharmacokinetic properties (i.e., absorption, metabolism, export) compared to those that elicit reporter expression.

Reviewer 1-Comment 4a: I accept the deferrals on ChIP-seq, motif analysis, heterodimerization, and identifying the Streptomyces metabolites. The claims should track them: the bacterial section supports NR-dependent induction by environmental bacteria, not yet an explanation for why the pathway evolved.
Our response: We have implemented this suggestion. We have changed the relevant sentence from, 'These observations suggest that a CAD defense pathway may have evolved in *C. elegans* to increases its survival rate in the presence of CAD-producing microbes.', to, 'Together, these observations suggest that a CAD defense pathway may help *C. elegans* survive in the presence of CAD-producing microbes.'

Reviewer 1-Comment 4b: please note that NR regulation of the targets is not shown to be direct;
Our response: Please see our response to this issue in the related responses to Reviewer 1-Comment 1 above.

Reviewer 1-Comment 4c: and please reframe "at least as good as a computational predictor," since the reporter recovers 68% of gold standards to the model's 73% on the same library the hits came from.
Our response: We have implemented this suggestion. We have changed the original sentence, 'Hence, our *in vivo* CAD defence reporter is at least as good as a computational predictor of CAD-induced pathologies in humans and provides a facile model for testing predictions.' to, 'Hence, our *in vivo* CAD defence reporter is a good *in vivo* predictor of CAD-induced pathologies in humans.'

Reviewer 1-Comment 5a: The one-tailed t-tests in Figures 5 and 6 need justification;
Our response: We use a one tailed test because we expected a trend in a particular direction in advance of data analyses, as articulated in the preamble in the relevant results section.

Reviewer 1-Comment 5b: The one-tailed t-tests in Figures 5 and 6 need multiple-comparison correction;
Our response: Each comparison we are making is a separate hypothesis that we are testing. For example, it is not examining many individuals within a population compared to a wild type control. We have multiple samples in these experiments, but they are distinct tests of different drugs or P450 alleles. If we removed all other samples except one experimental and one control, the validity of the test would remain the same. Hence, we do not see the value of a multiple-comparison correction.

Reviewer 1-Comment 5c: please state whether hit scoring was blinded and
Our response: We have implemented this suggestion. We have included the statement, 'No blinding was used in the experimental design' in the legend.

Reviewer 1-Comment 5d: why the myo-2p::mCherry control was dropped;
Our response: We apologize in advance, but we do not understand the issue here nor the experiment to which the reviewer is referring to.

Reviewer 1-Comment 5e: deposit the screen WGS and raw LCMS data.
Our response: We have deposited the original 39 WGS sequence files at NCBI and have included the accession numbers and links in DataFile 1. We have added the raw counts for the LCMS data in Figure 5 to DataFile 1 (there are 739 LCMS runs/files for the worm-CAD work, the counts of which are in Tab 13b; there are 841 LCMS runs/files for the yeast-CAD work, the counts of which are in Tab 14b).The raw counts for the LCMS data in Figure 6 are also in DataFile 1 in tab 15 (there are 142 LCMS runs/files associated with that set of experiments). The uploading of 1772 LCMS files and associated information for each one to a public repository would be unduly onerous, hence, we elected to show the raw counts of the relevant peaks from which we used to perform our calculations with in the DataFile.

Technical Requests

Request 1: Please ensure that the funders and grant numbers match between the Financial Disclosure field and the Funding Information tab in your submission form. Note that the funders must be provided in the same order in both places as well. Currently, " Canadian Institutes of Health Research Project Grant PJT-186117 (HMK), a Canada Research Chair (Tier 1) grant in Chemical Biology (PJR), an Ontario Graduate Scholarship to LT" are missing from the Funding Information and also the order is different in both the places.
Our response: We are confused on this point. When we look at the ‘add funding details box in the Financial Discolsure box, it is identical to what we have written in the Funding section of the manuscript (see screenshot below). We are probably missing something and need more explicit instructions.

Financial Disclosure

Enter a financial disclosure statement that describes the sources of funding for the work included in this submission. Review the submission guidelines for detailed requirements. View published research articles from *PLOS Biology* for specific examples.

This statement is required for submission and **will appear in the published article** if the submission is accepted. Please make sure it is accurate.

Funded studies
Enter a statement with the following details:

- Initials of the authors who received each award
- Grant numbers awarded to each author
- The full name of each funder
- URL of each funder website
- Did the sponsors or funders play any role in the study design, data collection and analysis, decision to publish, or preparation of the manuscript?

Did you receive funding for this work?

Answer Required: Yes

Please add funding details.

Answer Required: Character Count: 523

Funders include the Canadian Institutes of Health Research Project Grants 173448 and 186156 (PJR), Canadian Institutes of Health Research Project Grant PJT-186117 (HMK), a Canada Research Chair (Tier 1) grant in Chemical Biology (PJR), an Ontario Graduate Scholarship to LT, a

Limit 20000 characters

Request 2: We do not publish any copyright or trademark symbols that usually accompany proprietary names, eg (R), (C), or TM (e.g. next to drug or reagent names). Please remove all instances of trademark/copyright symbols throughout the text, including TRIzol™ ,Aurum™ TURBO™,Biosystems™, SsoAdvanced™, QuantStudio™, Anti-FLAG® on the manuscript file
Our response: We have removed all the ™s from the revised manuscript.

Request 3a: Please refer to the following article for PLOS Biology's best-practice recommendations for data presentation:
https://can01.safelinks.protection.outlook.com/?url=https%3A%2F%2Fjournals.plos.org%2Fplosbiology%2Farticle%3Fid%3D10.1371%2Fjournal.pbio.1002128&data=05%7C02%7Cpeter.roy%

40utoronto.ca%7Cb474677624d541ffa31508df019463ea%7C78aac2262f034b4d9037b46d56c55210%7C0%7C0%7C639231409003618427%7CUnknown%7CTWFpbGZsb3d8eyJFbXB0eU1hcGkiOnRydWUsIlYiOiIwLjAuMDAwMCIsIlAiOiJXaW4zMiIsIkFOIjoiTWFpbCIsIldUIjoyfQ%3D%3D%7C0%7C%7C%7C&sdata=at6Ek%2FMNjnl0VLdJuzNTUS24ZFSt3FVhbRmRmDBcMSk%3D&reserved=0.

Our response: Where bars have been used in our graphs, we have included the individual data points that represent the means of each independent biological trial.

Request 3b: Guidelines for statistical reporting, if relevant, may be found here: https://can01.safelinks.protection.outlook.com/?url=https%3A%2F%2Fjournals.plos.org%2Fplosbiology%2Fs%2Fbest-practices-in-research-reporting%23loc-statistical-reporting&data=05%7C02%7Cpeter.roy%40utoronto.ca%7Cb474677624d541ffa31508df019463ea%7C78aac2262f034b4d9037b46d56c55210%7C0%7C0%7C639231409003651816%7CUnknown%7CTWFpbGZsb3d8eyJFbXB0eU1hcGkiOnRydWUsIlYiOiIwLjAuMDAwMCIsIlAiOiJXaW4zMiIsIkFOIjoiTWFpbCIsIldUIjoyfQ%3D%3D%7C0%7C%7C%7C&sdata=j2PHuZoUklmRu6NpSOT5kfScYdLLu4y77XELLTdu1HQ%3D&reserved=0

Our response: We have done our best to comply with these guidelines.
