# Supplementary material for: The nematode Caenorhabditis elegans has a cationic amphiphilic drug (CAD) defense system
Source: PLoS Biol. 2026 Sep 21;24(9):e3004006. doi: 10.1371/journal.pbio.3004006 (PMC13619054; doi:10.1371/journal.pbio.3004006)
Supplement: Peer review history

# Editor report: Decision Letter 0

**DOI:** 10.1371/journal.pbio.3004006.r001

**Authors:** Ines Alvarez-Garcia

2 Feb 2026

Dear Dr Roy,

Thank you for submitting your manuscript entitled "A Cationic Amphiphilic Drug (CAD) Defense System in the Nematode Caenorhabditis elegans" for consideration as a Research Article by PLOS Biology.

Your manuscript has now been evaluated by the PLOS Biology editorial staff as well as by an academic editor with relevant expertise and I am writing to let you know that we would like to send your submission out for external peer review.

However, before we can send your manuscript to reviewers, we need you to complete your submission by providing the metadata that is required for full assessment. To this end, please login to Editorial Manager where you will find the paper in the 'Submissions Needing Revisions' folder on your homepage. Please click 'Revise Submission' from the Action Links and complete all additional questions in the submission questionnaire.

Once your full submission is complete, your paper will undergo a series of checks in preparation for peer review. After your manuscript has passed the checks it will be sent out for review. To provide the metadata for your submission, please Login to Editorial Manager (https://www.editorialmanager.com/pbiology) within two working days, i.e. by Feb 04 2026 11:59PM.

Feel free to email us at plosbiology@plos.org if you have any queries relating to your submission.

Kind regards,

Ines

--

Ines Alvarez-Garcia, PhD

Senior Editor

PLOS Biology

ialvarez-garcia@plos.org

# Peer review: Decision Letter 1

**DOI:** 10.1371/journal.pbio.3004006.r002

**Authors:** Ines Alvarez-Garcia

3 Apr 2026

Dear Dr Roy,

Thank you for your patience while your manuscript entitled "A Cationic Amphiphilic Drug (CAD) Defense System in the Nematode Caenorhabditis elegans" was peer-reviewed at PLOS Biology. Please also accept my apologies for the delay in sending you our decision. The manuscript has now been evaluated by the PLOS Biology editors, an Academic Editor with relevant expertise, and by three independent reviewers.

The reviews are attached below. As you will see, the reviewers find the conclusions novel and interesting, but they also raise several issues that will need to be addressed before we can consider the manuscript for publication. Reviewer 1 thinks that a ligand binding essay should be performed to confirm CAD binding to NHR-70 and NHR-107 to strengthen the study. This reviewer also requests an additional assay to confirm the experiments with lysosomal acidity using cDCFDA, among other issues. Reviewer 2 is mostly satisfied and only raises minor points, whereas Reviewer 3 notes that parts of the study are a bit hard to follow, thus the paper would benefit from some rewriting to make it more accessible for a wide audience. In addition, the reviewer suggests that a cartoon could be used to explain the model better.

In light of the reviews, which you will find at the end of this email, we would like to invite you to revise the work to thoroughly address the reviewers' reports. Given the extent of revision needed, we cannot make a decision about publication until we have seen the revised manuscript and your response to the reviewers' comments. Your revised manuscript is likely to be sent for further evaluation by all or a subset of the reviewers.

In addition to these revisions, you will need to complete some formatting changes, which you will receive in a follow up email. A member of our team will be in touch with a set of requests shortly.

We expect to receive your revised manuscript within 3 months. Please email us (plosbiology@plos.org) if you have any questions or concerns, or would like to request an extension.

At this stage, your manuscript remains formally under active consideration at our journal; please notify us by email if you do not intend to submit a revision so that we may withdraw it.

**IMPORTANT - SUBMITTING YOUR REVISION**

Your revisions should address the specific points made by each reviewer. Please submit the following files along with your revised manuscript:

1. A 'Response to Reviewers' file - this should detail your responses to the editorial requests, present a point-by-point response to all of the reviewers' comments, and indicate the changes made to the manuscript.

*NOTE: In your point-by-point response to the reviewers, please provide the full context of each review. Do not selectively quote paragraphs or sentences to reply to. The entire set of reviewer comments should be present in full and each specific point should be responded to individually, point by point.

You should also cite any additional relevant literature that has been published since the original submission and mention any additional citations in your response.

2. In addition to a clean copy of the manuscript, please also upload a 'track-changes' version of your manuscript that specifies the edits made. This should be uploaded as a "Revised Article with Changes Highlighted" file type.

3. Resubmission Checklist

When you are ready to resubmit your revised manuscript, please refer to this resubmission checklist: https://plos.io/Biology_Checklist

To submit a revised version of your manuscript, please go to https://www.editorialmanager.com/pbiology/ and log in as an Author. Click the link labelled 'Submissions Needing Revision' where you will find your submission record.

Please make sure to read the following important policies and guidelines while preparing your revision and fulfil the editorial requests:

a) *PLOS Data Policy*

Please note that as a condition of publication PLOS' data policy (http://journals.plos.org/plosbiology/s/data-availability) requires that you make available all data used to draw the conclusions arrived at in your manuscript. If you have not already done so, you must include any data used in your manuscript either in appropriate repositories, within the body of the manuscript, or as supporting information (N.B. this includes any numerical values that were used to generate graphs, histograms etc.). Please also indicate in each figure legend where the data can be found. For an example see here: http://www.plosbiology.org/article/info%3Adoi%2F10.1371%2Fjournal.pbio.1001908#s5

b) *Published Peer Review*

Please note while forming your response, if your article is accepted, you may have the opportunity to make the peer review history publicly available. The record will include editor decision letters (with reviews) and your responses to reviewer comments. If eligible, we will contact you to opt in or out. Please see here for more details:

https://blogs.plos.org/plos/2019/05/plos-journals-now-open-for-published-peer-review/

c) *Blot and Gel Data Policy*

Please provide the original, uncropped and minimally adjusted images supporting all blot and gel results reported in an article's figures or Supporting Information files. We will require these files before a manuscript can be accepted so please prepare them now, if you have not already uploaded them. Please carefully read our guidelines for how to prepare and upload this data: https://journals.plos.org/plosbiology/s/figures#loc-blot-and-gel-reporting-requirements

d) *Protocols deposition*

To enhance the reproducibility of your results, we recommend that if applicable you deposit your laboratory protocols in protocols.io, where a protocol can be assigned its own identifier (DOI) such that it can be cited independently in the future. For instructions see: https://journals.plos.org/plosbiology/s/submission-guidelines#loc-materials-and-methods

Thank you again for your submission to our journal. We hope that our editorial process has been constructive thus far, and we welcome your feedback at any time. Please don't hesitate to contact us if you have any questions or comments.

Sincerely,

Ines

--

Ines Alvarez-Garcia, PhD

Senior Editor

PLOS Biology

ialvarez-garcia@plos.org

------------------------------------

Reviewers' comments

Rev. 1:

This study identifies a defence system in Caenorhabditis elegans that protects against cationic amphiphilic drugs (CADs), which can cause lysosomal dysfunction. Using a combination of genetics, transcriptomics, metabolomics, chemoinformatics, and structural modeling, the authors provide compelling evidence that nuclear receptors NHR 70 and NHR 107 regulate CYP 35B enzymes and PGP 13/PGP 14 transporters to detoxify CADs. The authors provide evidence for CYP 35Bs metabolizing CADs and PGP 13/14 exporting metabolites, both critical for reducing drug-induced toxicity. A pgp 13 fluorescent reporter enabled them to screen a compound library, identifying CAD-like compounds consistent with computational predictions. They show that the defence system is also activated by certain Streptomyces strains, hinting at its evolutionary purpose. While some mechanisms remain unclear, in general, the study is well designed and executed, have several elements of novelty and will be of broad interest to the scientific community.

Comments:

1. One of the main drawbacks of the study is the lack of direct evidence of CAD binding to NHR-70 and NHR-107. Direct evidence in form of a ligand binding assay would strengthen their study.

2. Does NHR-70 and NHR-107 directly regulate the expression of the pgp genes?

3. 76% of compounds require both NHR-70 and NHR-107 while 12% require only NHR-70. This suggests a potential combinatorial regulation. Do NHR-70 and NHR-107 form heterodimers?

4. The relevance of the last section of the manuscript that deals with checking the response to Actinobacteria strains is quite vague. Out of the 65 strains, only two showed obvious increase in the pgp-13::gfp expression while all these strains are claimed to produce secondary metabolites. What are the metabolites produced by the strains 1G9 and 1E6 that induce the GFP?

5. The experiments with lysosomal acidity using cDCFDA should be supported by another assay (may refer https://doi.org/10.52601/bpr.2021.200047 or similar studies).

6. The authors claim, "Consistent with this interpretation, many of the gold standards that failed to induce pgp-13 expression have EC50 concentrations for PLD-induction in human cells that far exceed the concentration at which we screened in worms (30 μM) (e.g., amantadine, memantine, spectinomycin (MORELLI et al. 2006)". They need to show in the main text that higher concentrations would induce pgp-13:gfp.

7. In Figure 7B and C, single and double of pgp-13 and pgp-14 should be added. Are they redundant?

Rev. 2:

Overall comments:

This study is a very nice combination of genetic, computational, biochemical, and ecological/evolutionary approaches. It addresses an interesting question from a drug discovery and safety perspective, and contributes impactfully to the field's very early-stage understanding of xenobiotic metabolism pathways in C. elegans. It is quite a lot of rigorously-done work, and clearly and fairly interpreted and discussed. It is clearly written and well-organized. I am not sure I have ever had so little to say in >25 years of reviewing manuscripts. Thank you!

Specific comments:

Figure 1 legend: "Three independent repeats were performed (N>3); an upper ceiling of 50 animals per sample were counted"—I think this should be "N = 3"? and please indicate also the lower range of animals per sample counted.

I recommend including a metadata file in the first tab of the Supplementary excel file.

Page 17 "dependent" typo: "We tested whether this increase in signal was dependant on NHR-70 and NHR-107 and found that it was (p<0.005)"

Rev. 3:

This is an interesting piece of work that uses an extensive range of experimental approaches in C elegans to probe mechanisms of xenobiotic detoxification. The take home message seems to be that the nematodes can mount a defence against CADs - this is a novel finding as I understand that such a mechanism has not been identified in other organisms. The most exciting prospect would be that these systems do exist in human but are as yet undiscovered with the possibility that this work might provide a route in.

The starting point for the study is a bit hard to follow. Is it really about PGP-14 as a model for human ABC4 deficiency? The paper writes about the pgp-14 phenotype - is this constrained to the resistance to wact-190? Or is it more extensive? If it's really about ABC4 and pgp-14 why not conduct a complementation experiment? i.e. can ABCB4 rescue pgp-14?

But rather I think that their screen has provided an interesting way to reveal the interaction between PGP-13, PGP-14, upstream NHR regulation and CAD metabolism.

If I understand correctly, the authors propose that in the pgp-14 ko CADS sensitize the worm to wact-190 because they upregulate pgp-13, which can then perform the same function in terms of promoting crystal formation. Furthermore, they reveal upstream regulation of this phenomenon. They then show that pgp-13 ko, like pgp-14 ko, can shift the dose-response for CAD lethality. I would like to suggest the authors rewrite some of the framing of this work to make it more 'digestible'. A cartoon would really help with the message.

Some more specific comments:

Why no further comment on tat-3?

Could the authors comment more explicitly on the baseline effect of the CADs in the study on wild-type? And how the dosing was chosen for the pgp-14 ko screen in this regard?

Fig 7. How are the worms identified as dead? Is it possible they are just immobilised? What was the stage of the worm? Adult? What was the exposure time?

Typos; page 9, 17 and possibly elsewhere- 'dependant' should be 'dependent'

Fig 1 legend D. should this really be 30mM?

P 23 typo 'Hence, we endeavored To'

# Author comment: Author response to Decision Letter 2

**DOI:** 10.1371/journal.pbio.3004006.r003

4 Jul 2026

Attachment Submitted filename: CADs-Response to Reviews & Appendices-Final.pdf

# Peer review: Decision Letter 2

**DOI:** 10.1371/journal.pbio.3004006.r004

**Authors:** Ines Alvarez-Garcia

21 Aug 2026

Dear Dr Roy,

Thank you for your patience while we considered your revised manuscript entitled "A Cationic Amphiphilic Drug (CAD) Defense System in the Nematode Caenorhabditis elegans" for publication as a Research Article at PLOS Biology. This revised version of your manuscript has been evaluated by the PLOS Biology editors, the Academic Editor and one of the original reviewers.

Based on the comments (attached below), we are likely to accept this manuscript for publication, provided you satisfactorily address the remaining points raised by Reviewer 1. After consulting with the Academic Editor, we agree that all the negative results should be reported in the manuscript, but that the statement that "CADs may directly interact with the nuclear receptors" is fine. In point 4, you could replace "at least as good as" with "a good predictor" as 78% vs 73% is in the margin of error. You could also say that "C. elegans may increase its survival rate in the presence of CAD-producing microbes." Please also make sure to address the policy-related requests stated below my signature.

In addition, we would like you to consider a suggestion to improve the title:

"C. elegans have a cationic amphiphilic drug (CAD) defense system”

As you address these items, please take this last chance to review your reference list to ensure that it is complete and correct. If you have cited papers that have been retracted, please include the rationale for doing so in the manuscript text, or remove these references and replace them with relevant current references. Any changes to the reference list should be mentioned in the cover letter that accompanies your revised manuscript.

In addition to these revisions, you may need to complete some formatting changes, which you will receive in a follow up email. A member of our team will be in touch with a set of requests shortly. If you do not receive a separate email within a few days, please assume that checks have been completed, and no additional changes are required.

We expect to receive your revised manuscript within two weeks.

To submit your revision, please go to https://www.editorialmanager.com/pbiology/ and log in as an Author. Click the link labelled 'Submissions Needing Revision' to find your submission record. Your revised submission must include the following:

- a cover letter that should detail your responses to any editorial requests, if applicable, and whether changes have been made to the reference list

- a Response to Reviewers file that provides a detailed response to the reviewers' comments (if applicable, if not applicable please do not delete your existing 'Response to Reviewers' file.)

- a track-changes file indicating any changes that you have made to the manuscript.

NOTE: If Supporting Information files are included with your article, note that these are not copyedited and will be published as they are submitted. Please ensure that these files are legible and of high quality (at least 300 dpi) in an easily accessible file format. For this reason, please be aware that any references listed in an SI file will not be indexed. For more information, see our Supporting Information guidelines:

https://journals.plos.org/plosbiology/s/supporting-information

*Published Peer Review History*

Please note that you may have the opportunity to make the peer review history publicly available. The record will include editor decision letters (with reviews) and your responses to reviewer comments. If eligible, we will contact you to opt in or out. Please see here for more details:

https://plos.org/published-peer-review-history/

*Press*

Should you, your institution's press office or the journal office choose to press release your paper, please ensure you have opted out of Early Article Posting on the submission form. We ask that you notify us as soon as possible if you or your institution is planning to press release the article.

*Protocols deposition*

To enhance the reproducibility of your results, we recommend that if applicable you deposit your laboratory protocols in protocols.io, where a protocol can be assigned its own identifier (DOI) such that it can be cited independently in the future. Additionally, PLOS ONE offers an option for publishing peer-reviewed Lab Protocol articles, which describe protocols hosted on protocols.io. Read more information on sharing protocols at https://plos.org/protocols?utm_medium=editorial-email&utm_source=authorletters&utm_campaign=protocols

Please do not hesitate to contact me should you have any questions.

Sincerely,

Ines

--

Ines Alvarez-Garcia, PhD

Senior Editor

PLOS Biology

ialvarez-garcia@plos.org

------------------------------------------------------------------------

DATA POLICY:

Thank you for providing the data underlying all the graphs shown in the figures. Please ensure that the data file is named using the following format verbatim: S1 Data, and that is saved using exactly the following convention: S1_Data.xlsx (using an underscore).

In addition, please make sure that this file is mentioned in the figure legends of all the corresponding figures to state where the data can be found.

------------------------------------------------------------------------

CODE POLICY

Per journal policy, if you have generated any custom code during the course of this investigation, please make it available without restrictions. Please ensure that the code is sufficiently well documented and reusable, and that your Data Statement in the Editorial Manager submission system accurately describes where your code can be found. More information on our Code Policy, what and how to share can be found here: https://journals.plos.org/plosbiology/s/code-availability

Please note that we cannot accept sole deposition of code in GitHub, as this could be changed after publication. However, you can archive this version of your publicly available GitHub code to Zenodo. Once you do this, it will generate a DOI number, which you will need to provide in the Data Accessibility Statement (you are welcome to also provide the GitHub access information). See the process for doing this here: https://docs.github.com/en/repositories/archiving-a-github-repository/referencing-and-citing-content

------------------------------------------------------------------------

Reviewers' comments

Rev. 1:

I thank the authors for the extensive new work and for a rebuttal that is candid about experiments that did not succeed. My concern is narrow: four experiments were done at my request, all returned negative results, and none appear in the manuscript.

1. Three approaches — GAL4-LBD/luciferase in HepG2, AlphaFold3/Boltz-2 docking with PXR and VDR benchmarks, and LBD pull-downs with LC-MS, all returned negative results but are not reported in the manuscript; a reader would never know about these. "Remains an open question" no longer fairly describes what you know. Please add Appendices A and B as supplemental figures with brief text, including your caveats about absent worm-specific cofactors. New Figure 9 opens with "CADs may directly interact with the nuclear receptors." Please give the two arms of the model equal weight and cite the negative data in the legend.

2. pHTomato showed no change in response to any CAD or to 10 mM chloroquine. The failed positive control indicates that the assay was uninformative for intestinal LROs; this is worth publishing, as it will spare others the effort. Please report Figure R1. cDCFDA therefore remains the sole pathology readout, and phospholipidosis itself is still never measured. Please state in the Discussion that the relationship to PLD is inferred.

3. Amantadine, memantine and spectinomycin were tested at higher concentrations and none induced (Figure R2). The revision replaces the original claim with an uncheckable restatement of the same permeability hypothesis while the disconfirming data stay unpublished. Please include Figure R2 and state in the Results that the concentration explanation does not hold.

4. I accept the deferrals on ChIP-seq, motif analysis, heterodimerization, and identifying the Streptomyces metabolites. The claims should track them: the bacterial section supports NR-dependent induction by environmental bacteria, not yet an explanation for why the pathway evolved; please note that NR regulation of the targets is not shown to be direct; and please reframe "at least as good as a computational predictor," since the reporter recovers 68% of gold standards to the model's 73% on the same library the hits came from.

5. The one-tailed t-tests in Figures 5 and 6 need justification and multiple-comparison correction; please state whether hit scoring was blinded and why the myo-2p::mCherry control was dropped; and deposit the screen WGS and raw LCMS data.

In summary, the genetics is carefully conducted, the necessity/sufficiency case for the CYP-35Bs is convincing, and the reporter plus annotated compound set is a resource the field will use. But as it stands, the sensing step remains unresolved and now has three negative datasets against it; the pathology in the title is measured only by proxy; and the reporter's translational claim rests on a comparison whose main caveat has been experimentally excluded.

Without the additional validation described in points 1-3, I do not think the work supports the breadth of claim expected for this venue. It would be better suited to a specialist journal, such as PLOS Genetics or Genetics, where the negative results and limitations could be reported fully and would make a solid, useful contribution. I leave the broader question of general-interest significance to the editors, who are better placed to weigh it alongside the other reviewers' assessments.

# Author comment: Author response to Decision Letter 3

**DOI:** 10.1371/journal.pbio.3004006.r005

1 Sep 2026

Attachment Submitted filename: CAD-response to second round reviews-1f.pdf

# Editor report: Decision Letter 3

**DOI:** 10.1371/journal.pbio.3004006.r006

**Authors:** Ines Alvarez-Garcia

4 Sep 2026

Dear Dr Roy,

Thank you for the submission of your revised Research Article entitled "The Nematode Caenorhabditis elegans has a Cationic Amphiphilic Drug (CAD) Defense System" for publication in PLOS Biology. On behalf of my colleagues and the Academic Editor, Mark Alkema, I am delighted to let you know that we can in principle accept your manuscript for publication, provided you address any remaining formatting and reporting issues. These will be detailed in an email you should receive within 2-3 business days from our colleagues in the journal operations team; no action is required from you until then. Please note that we will not be able to formally accept your manuscript and schedule it for publication until you have completed any requested changes.

Please take a minute to log into Editorial Manager at http://www.editorialmanager.com/pbiology/, click the "Update My Information" link at the top of the page, and update your user information to ensure an efficient production process.

PRESS

We frequently collaborate with press offices. If your institution or institutions have a press office, please notify them about your upcoming paper at this point, to enable them to help maximise its impact. If the press office is planning to promote your findings, we would be grateful if they could coordinate with biologypress@plos.org. If you have previously opted in to the early version process, we ask that you notify us immediately of any press plans so that we may opt out on your behalf.

We also ask that you take this opportunity to read our Embargo Policy regarding the discussion, promotion and media coverage of work that is yet to be published by PLOS. As your manuscript is not yet published, it is bound by the conditions of our Embargo Policy. Please be aware that this policy is in place both to ensure that any press coverage of your article is fully substantiated and to provide a direct link between such coverage and the published work. For full details of our Embargo Policy, please visit http://www.plos.org/about/media-inquiries/embargo-policy/.

Many congratulations and thanks again for choosing PLOS Biology for publication and supporting Open Access publishing. We look forward to publishing your study.

Sincerely,

Ines

--

Ines Alvarez-Garcia, PhD

Senior Editor

PLOS Biology

ialvarez-garcia@plos.org
